# Synthesis of Benzylidene Analogs of Oleanolic Acid as Potential *α*-Glucosidase and *α*-Amylase Inhibitors

**DOI:** 10.3389/fchem.2022.911232

**Published:** 2022-06-08

**Authors:** Jun-Jie Ke, Jing Lin, Xin Zhang, Xiao-Zheng Wu, Ying-Ying Zheng, Chun-Mei Hu, Yu Kang, Kun Zhang, Zhuang Xiong, Zhi-Qiang Ma

**Affiliations:** School of Biotechnology and Health Sciences, Wuyi University, Jiangmen, China

**Keywords:** oleanolic acid, *α*-amylase, *α*-glucosidase, enzyme inhibitor, docking

## Abstract

A series of benzylidene analogs of oleanolic acid **4a∼4s** were synthesized and assessed for their *α*-glucosidase and *α*-amylase inhibitory activities. The results presented that all synthesized analogs exhibited excellent-to-moderate inhibitory effects on *α*-glucosidase and *α*-amylase. Analog **4i** showed the highest *α*-glucosidase inhibition (IC_50_: 0.40 μM), and analog **4o** presented the strongest *α*-amylase inhibition (IC_50_: 9.59 μM). Inhibition kinetics results showed that analogs **4i** and **4o** were reversible and mixed-type inhibitors against *α*-glucosidase and *α*-amylase, respectively. Simulation docking results demonstrated the interaction between analogs and two enzymes. Moreover, analogs **4i** and **4o** showed a high level of safety against 3T3-L1 and HepG2 cells.

## Introduction

Type 2 diabetes mellitus (T2DM) is a metabolic disease characterized by hyperglycemia resulting from insulin resistance and insufficient insulin secretion by pancreatic β-cells (Silva et al., 2016; Ahmed et al., 2020). T2DM can also bring about complications such as hepatic, cardiac, and renal disorders (Forouhi and Wareham, 2010; Mollica et al., 2018). In addition, the aggravation of insulin resistance and pancreatic β-cell dysfunction can be ascribed to genetic predisposition, increasing age, and obesity (Kokil et al., 2015; Mollica et al., 2017). It is conservatively estimated that T2DM will affect approximately 500 million people worldwide by 2030, and the mortality rate for the disease and its associated complications is one death every 6 s as of now (Kumar et al., 2021). The effective way to reduce blood glucose levels in the treatment and prevention of T2DM and its complications is the clinical use of oral hypoglycemic agents such as sulfonylureas (Liang et al., 2020; Ling et al., 2021), biguanides (Zheng T.-L. et al., 2021; Kathuria et al., 2021), thiazolidinedione-derived drugs (Long et al., 2021; Liu et al., 2021), dipeptidyl-peptidase IV inhibitors (Carullo et al., 2021; Cheng et al., 2022), *α*-glucosidase inhibitors (Hossain et al., 2020), sodium-glucose cotransporter-2 inhibitors (Provenzano et al., 2021), and glucagon-like peptide-1 (GLP-1) receptor agonists (Furer et al., 2021; Saxena et al., 2021). Among these, *α*-glucosidase inhibitors are widely used as clinical drugs including acarbose, voglibose, miglitol, and emiglitate, and there are reports on *α*-amylase inhibitors derived from the natural product. In addition, it is well-known that starch is hydrolyzed by *α*-amylases into disaccharides or oligosaccharides in the mouth and small intestine, respectively, followed by the further hydrolysis of *α*-glucosidases into glucose units in the small intestinal lumen. The inhibitors against *α*-glucosidases or *α*-amylases can combine with the active units of these two enzymes to form complexes with stronger affinity than that of the carbohydrate–enzyme complex, thereby realizing the inhibition against *α*-glucosidases or *α*-amylases. (Proença et al., 2021).

Oleanolic acids (OA) is a pentacyclic oleanane-type triterpenoid with a broad spectrum of bioactivities in which the potential application in the management of T2DM and its associated comorbidities, resulting from their antihyperglycemic, antihyperlipidemic, antiatherogenic, antioxidant, and anti-inflammatory action, has attracted much attention (Liu, 2005; Pollier and Goossens, 2012; Loza-Rodriguez et al., 2020). It is worth noting that OA can reduce postprandial hyperglycemia in diabetic people by inhibiting *α*-glucosidase and the pancreatic and salivary *α*-amylase without apparent hepatotoxicity in the experimental studies (Castellano et al., 2013). Given that, much effort has been focused on the modification of OA in order to improve its potential druggability. So far, OA derivatives are mainly obtained by means of OA semi-synthetic pathways: 1) esterification, glycosylation, or oxidization at C-3 position (Jensen et al., 1997; Wu, et al., 2021; Zhong et al., 2021); 2) amidation or esterification at C-28 position (Tang et al., 2014; Kazakova et al., 2021); 3) lactonization between C-12 and C-28 positions (Zhong et al., 2019); and 4) condensation with various aldehydes at C-2 position (Zhong et al., 2019); all these derivatizations toward OA can improve *α*-glucosidase or *α*-amylase inhibitory activity. In addition, the benzylidene group is reported to be one key substituent in many pharmacological compounds, such as chalcones and cinnamic acids(Csuk, et al., 2012; Kim et al., 2014; Gupta et al., 2017), and the introduction of the benzylidene group can increase the inhibitory activity against *α*-glucosidase or *α*-amylase inhibitory activity in our previous work (Deng et al., 2022).

In view of these findings, the strategy to incorporate the benzylidene side chain into the OA skeleton was adopted to enhance the bioavailability of OA. Herein, the synthesis of benzylidene analogs **3a–s** of OA by Claisen–Schmidt condensation was developed, followed by reduction at the C-3 position to obtain OA analogs **4a–4s**. The *α*-glucosidase and *α*-amylase inhibitory activity of OA analogs has been evaluated *in vitro*. The results showed that most compounds revealed a better inhibitory effect than OA. On this basis, the inhibitory mechanism that OA analogs interacted with these two enzymes has been probed into by biochemical and computational assays.

## Results and Discussion

### Chemistry

**SCHEME 1:**
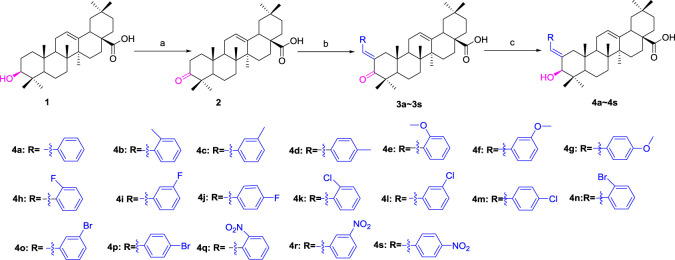
Synthesis of benzylidene analogs of oleanolic acid **4a**–**4s**. Reagent and condition: (a) Jones reagent, acetone, 0°C; (b) Substituted aromatic aldehydes, EtOH, KOH, room temperature, and overnight; (c) NaBH_4_, DCM, MeOH, 2h, 0 C—room temperature

OA was used as a starting material to obtain benzylidene analogs of oleanolic acid according to the synthetic route shown in Scheme 1. First, OA (**1**) was oxidized to 3-oxo-olean-12-en-28-oic acid (**2**) by Jones reagent, further reacting with substituted aromatic aldehydes to produce intermediate **3**. Then, target analogs **4a–4s** were prepared through reduction reaction of intermediate **3**. All synthetic analogs were identified by ^1^H NMR, ^13^C NMR, and HRMS.

### Inhibitory Effect of OA Analogs Against *α*-Glucosidase and *α*-Amylase

OA analogs **4a–4s** were first assessed for their *α*-glucosidase inhibitory activities, and the results are listed in Table 1. All synthesized OA analogs (**4a–4s**) exhibited potent *α*-glucosidase inhibitory activity (IC_50_: 0.40–3.96 μM), which was higher than that of OA (IC_50_: 4.09 μM). Analog **4i** showed the strongest *α*-glucosidase inhibition (IC_50_: 0.40 μM), which was ∼1,663 times stronger than that of acarbose (IC_50_: 665.56 μM). The results showed that the modification of OA with benzylidene could improve its *α*-glucosidase inhibitory activity. From the *α*-glucosidase inhibitory activities of OA analogs (**4a–4s**), it could be seen that introduction of the donating groups (methyl and methoxy) at the para-position of substituted aryl aldehydes could reduce inhibitory activities, while the introduction of the withdrawing group (fluorine, chlorine, bromine, and nitro) at the para-position could improve the inhibition. In addition, it was surprisingly found that the inhibition of compounds with substituents at meta-position was mostly superior to that of compounds with substituents at ortho-position, and the inhibition of compounds with substituents at the ortho-position was better than that of compounds with substituents at para-position. Finally, we found that analog **4i** with the withdrawing group (fluorine) at the meta-position of substituted aryl aldehydes showed the highest *α*-glucosidase inhibition (IC_50_: 0.40 μM).

**TABLE 1:** Inhibition of OA analogs (**4a**–**4s**) on *α*-glucosidase and *α*-amylase.

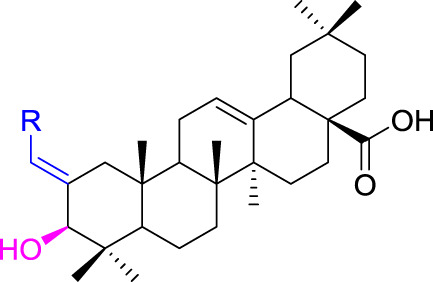			
**Compound**	**R**	***α*-glucosidase inhibition (IC_50_ μM)**	***α*-amylase inhibition (IC_50_ μM)**
**4a**	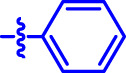	1.90 ± 0.31^a^ ^,^ ^b^	41.23 ± 2.99^a^ ^,^ ^b^
**4b**	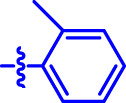	0.61 ± 0.09^a^ ^,^ ^b^	19.80 ± 1.22^a^ ^,^ ^b^
**4c**	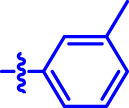	0.86 ± 0.05^a^ ^,^ ^b^	51.12 ± 2.55^a^ ^,^ ^b^
**4d**	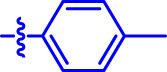	2.41 ± 0.08^a^ ^,^ ^b^	62.18 ± 1.89^a^ ^,^ ^b^
**4e**	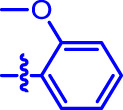	1.92 ± 0.13^a^ ^,^ ^b^	20.53 ± 2.29^a^ ^,^ ^b^
**4f**	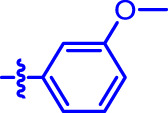	1.63 ± 0.10^a^ ^,^ ^b^	60.27 ± 1.88^a^ ^,^ ^b^
**4g**	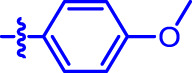	3.96 ± 0.21^a^ ^,^ ^b^	59.09 ± 1.98^a^ ^,^ ^b^
**4h**	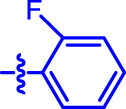	0.83 ± 0.04^a^ ^,^ ^b^	18.52 ± 1.39^a^ ^,^ ^b^
**4i**	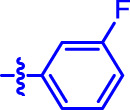	0.40 ± 0.02^a^ ^,^ ^b^	20.27 ± 2.18^a^ ^,^ ^b^
**4j**	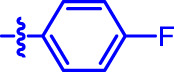	1.23 ± 0.07^a^ ^,^ ^b^	15.68 ± 1.53^a^ ^,^ ^b^
**4k**	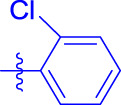	0.88 ± 0.04^a^ ^,^ ^b^	45.41 ± 2.16^a^ ^,^ ^b^
**4l**	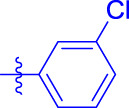	0.45 ± 0.02^a^ ^,^ ^b^	19.39 ± 0.73^a^ ^,^ ^b^
**4m**	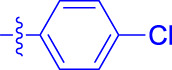	1.73 ± 0.16^a^ ^,^ ^b^	20.49 ± 1.09^a^ ^,^ ^b^
**4n**	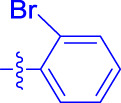	0.72 ± 0.06^a^ ^,^ ^b^	16.75 ± 1.04^a^ ^,^ ^b^
**4o**	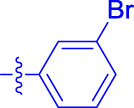	0.52 ± 0.02^a^ ^,^ ^b^	9.59 ± 0.58^a^ ^,^ ^b^
**4p**	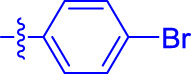	1.18 ± 0.14^a^ ^,^ ^b^	19.51 ± 1.85^a^ ^,^ ^b^
**4q**	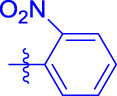	1.17 ± 0.10^a^ ^,^ ^b^	20.20 ± 1.20^a^ ^,^ ^b^
**4r**	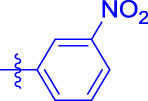	1.08 ± 0.04^a^ ^,^ ^b^	20.77 ± 1.88^a^ ^,^ ^b^
**4s**	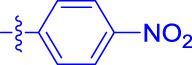	1.22 ± 0.14^a^ ^,^ ^b^	55.56 ± 2.52^a^ ^,^ ^b^
**OA**		4.09^a^	94.10^a^
**Acarbose**		665.56^b^	100.01^b^

a Indicating comparisons between the compound group with the **OA** group (*P* < 0.05)

b Indicating comparisons between the compound and **OA** groups with the **acarbose** group (*P* < 0.05).

Subsequently, OA analogs (**4a–4s**) were evaluated for their *α*-amylase inhibitory activities (Table 1). It was found that all OA analogs presented obvious *α*-amylase inhibitory activities with IC_50_ values of 9.59–65.78 μM, compared with those of OA (IC_50_: 94.10 μM) and analog **4o** had the strongest *α*-amylase inhibitory activity (IC_50_: 9.59 μM), which was ∼10 times higher than that of acarbose (IC_50_: 100.01 μM). The modification of OA with benzylidene could also contribute to the improvement of *α*-amylase inhibitory activity. But, the compound’s inhibitory activity has no relation with the electrical properties of substituents. Compared with the unsubstituted compound **4a**, introduction of functional groups (fluorine, chlorine, and bromine) at the C2, C3, and C4 position of the substituted aldehydes, functional groups (methyl and methoxy) at the C2 position, and nitro group at the C3 position of benzene group improved the inhibitory activity. But, the introduction of the donating groups (methyl and methoxy) at the C3 and C4 position and nitro group at the para-position of substituted aryl aldehydes resulted in lower inhibitory activity.

As could be seen from the aforementioned results, all OA analogs (**4a–4s**) displayed bifunctionality against *α*-glucosidase and *α*-amylase. All OA analogs displayed dual-inhibitory activities against *α*- glucosidase and *α*-amylase, which were better than those of acarbose.

### Inhibition Kinetics Assay Against *α*-Glucosidase and *α*-Amylase

**FIGURE 1:**
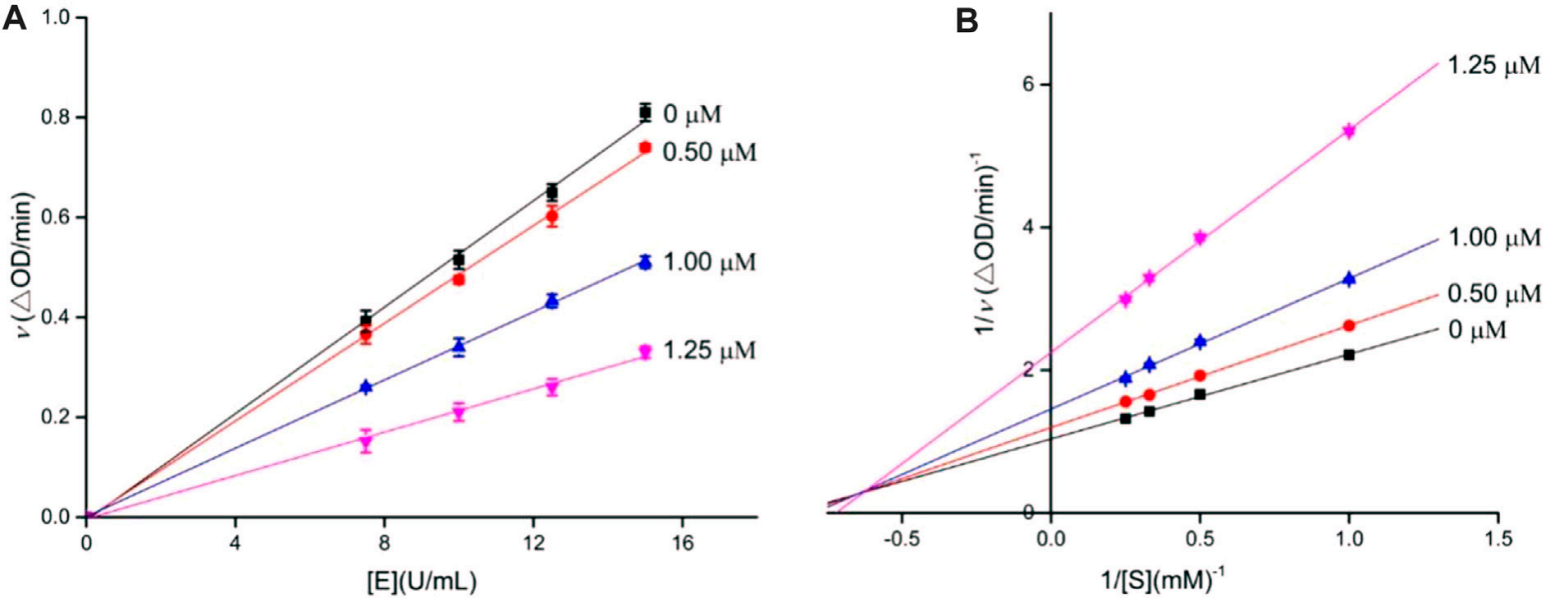
Inhibition kinetics of analog **4i** against *α*-glucosidase. **(A)** Plots of the enzymatic reaction rate *vs. α*-glucosidase concentration with or without the presence of analog **4i**; **(B)** Lineweaver–Burk plots of enzymatic reaction rate *vs*. substrate concentration with or without the presence of analog **4i**.

To decide the inhibition kinetics of OA analogs (**4a–4s**) against *α*-glucosidase and *α*-amylase, analogs **4i** and **4o** with the strongest *α*-glucosidase and *α*-amylase inhibitory activity, respectively, were selected as the representative analogs. As shown in Figure 1A, each trend line of the enzymatic reaction rate *vs. α*-glucosidase concentration with or without the presence of analog **4i** passed through the origin, showing that the inhibition of analog **4i** on *α*-glucosidase was reversible. Its inhibition kinetics parameters were assayed using the Lineweaver–Burk plots (Figure 1B). It could be seen that each trend line of the enzymatic reaction rate *vs*. substrate concentration with or without the presence of analog **4i** intersected in the second quadrant, indicating that analog **4i** was a mixed-type inhibitor against *α*-glucosidase.

**FIGURE 2:**
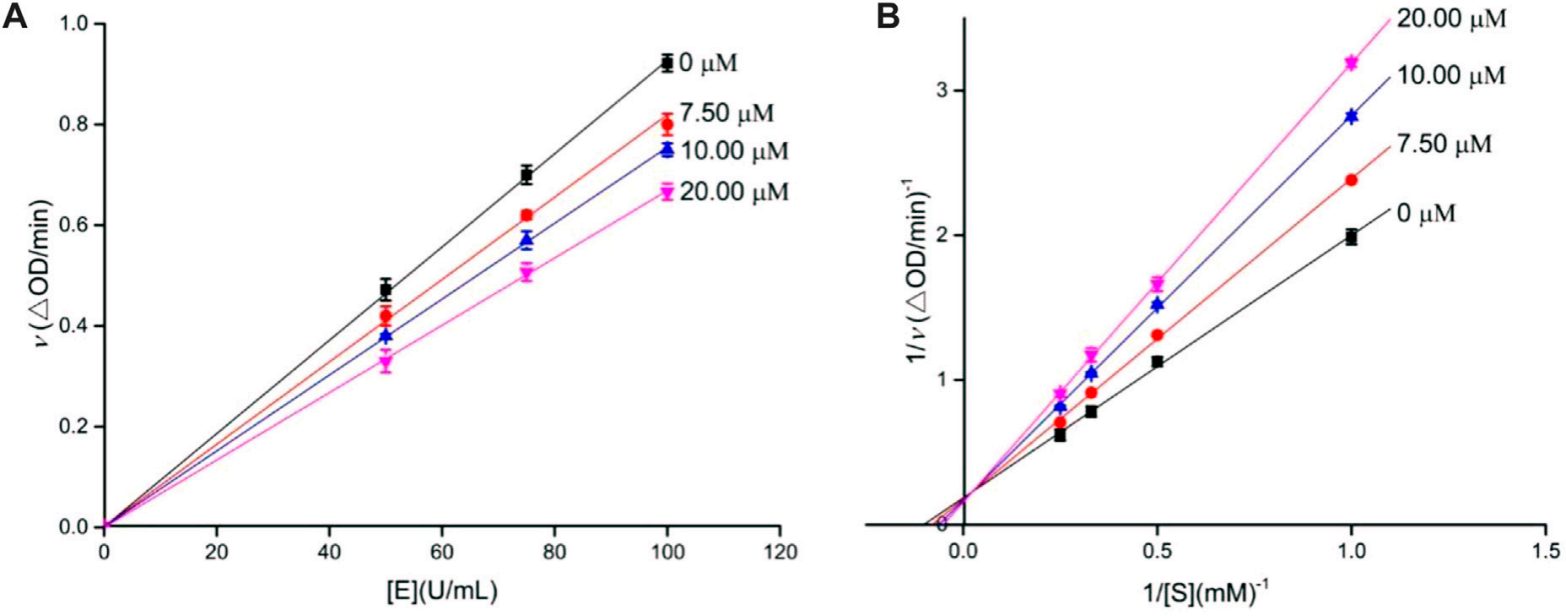
Inhibition kinetics of analog **4o** against *α*-amylase. **(A)** Plots of enzymatic reaction rate *vs. α*-amylase concentration with or without the presence of analog **4o**; **(B)** Lineweaver–Burk plots of enzymatic reaction rate *vs*. substrate concentration with or without the presence of analog **4o**.

Similarly, the inhibition kinetics of analog **4o** against *α*-amylase was studied. The plots of the enzymatic reaction rate *vs. α*-amylase concentration passed through the origin (Figure 2A), and Lineweaver–Burk plots of enzymatic reaction rate *vs*. substrate concentration intersected in the first quadrant (Figure 2B). The results showed that analog **4o** functioned as a reversible and mixed-type inhibitor against *α*-amylase.

### Molecular Docking Simulation

**FIGURE 3:**
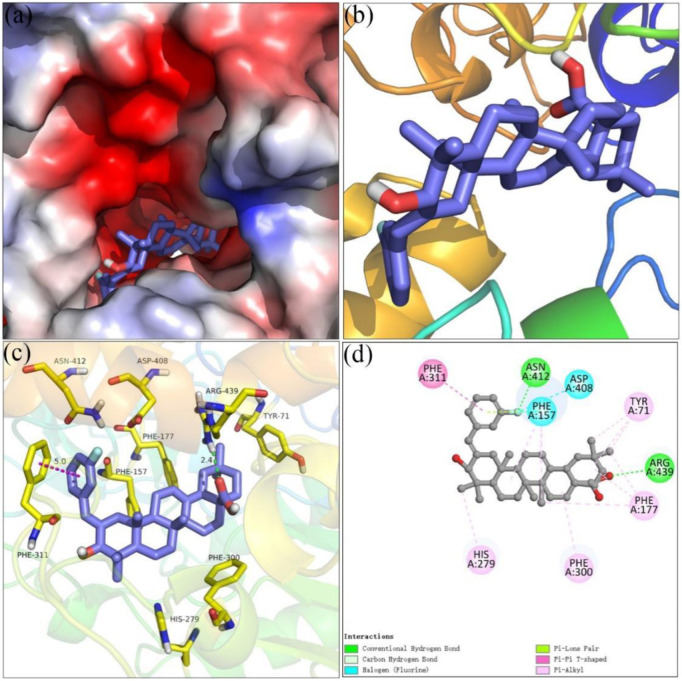
Molecular docking of analog **4i** with *α*-glucosidase. **(A) 4i** in the electrostatics active pocket; **(B) 4i** in the active pocket; **(C)** 3D view of **4i** with *α*-glucosidase; **(D)** 2D view of **4i** with *α*-glucosidase.

Molecular docking simulations using SYBYL software for analogs **4i** and **4o** were carried out targeting *α*-glucosidase and *α*-amylase, respectively. On the basis of docking results, the binding interactions were analyzed. The docking simulation between analog **4i** and homology model *α*-glucosidase is shown in Figure 3. Benzylidene and the hydroxyl moiety of analog **4i** bind at the entrance of the *α*-glucosidase pocket and carboxyl moiety positioned in the interior of the pocket (Figures 3A,B). Detailed analysis (Figures 3C,D) presented that the carboxyl moiety of analog **4i** formed a hydrogen bond with Arg 439 (2.4 Å). The fluorine substituent of benzylidene formed a hydrogen bond with Asn 412 and two halogen bonds with Phe 157 and Asp 408. The benzylidene formed a π–π stacking with Phe 311 (5.0 Å). Furthermore, analog **4i** formed hydrophobic interactions with Tyr 71, Phe 177, His 279, and Phe 300.

**FIGURE 4:**
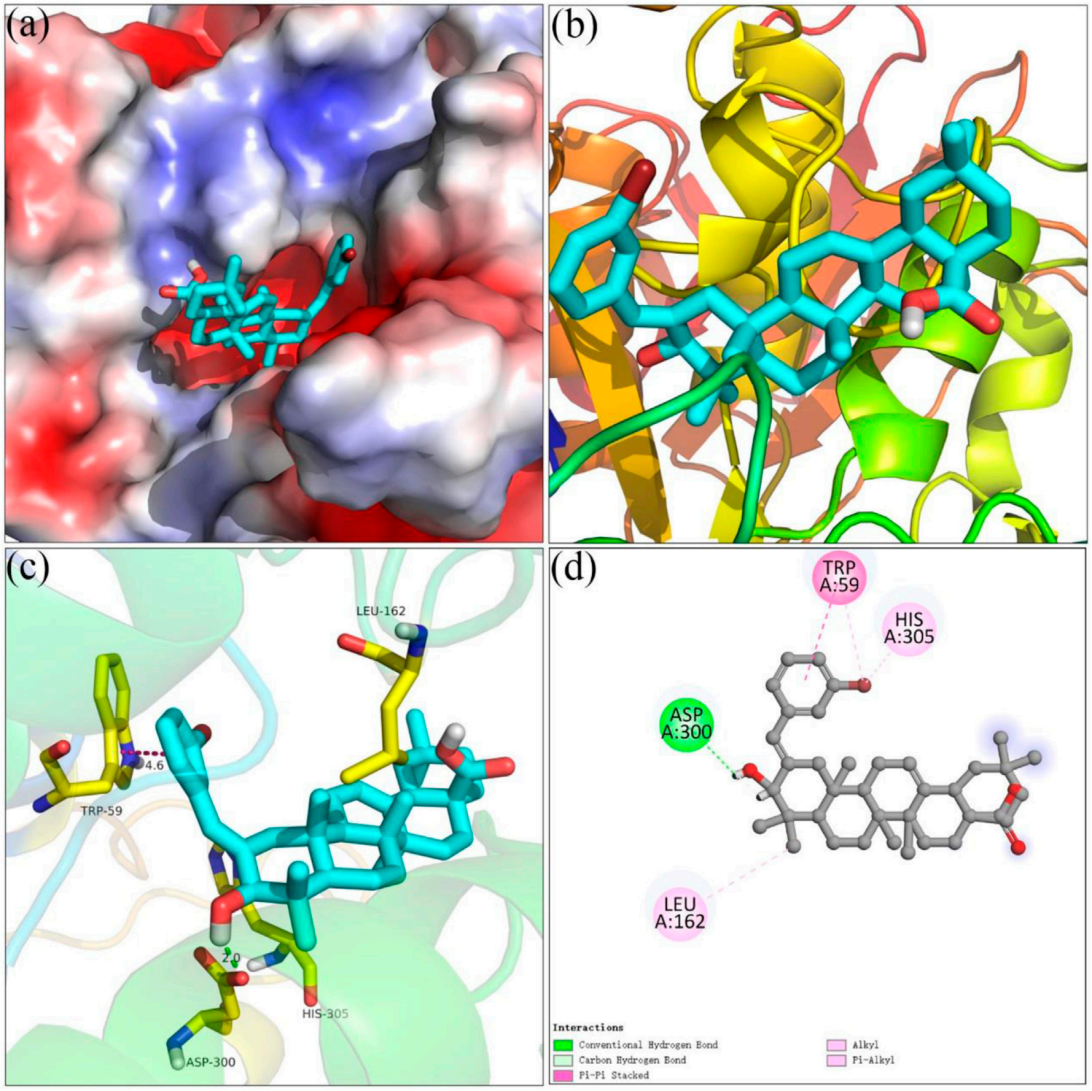
Molecular docking of analog **4o** and *α*-amylase. **(A) 4o** in the electrostatics active pocket; **(B) 4o** in the active pocket; **(C)** 3D view of **4o** and *α*-amylase; **(D)** 2D view of **4o** and *α*-amylase.

Figure 4 showed the docking results of analog **4o** and *α*-amylase. It could be seen that benzylidene and the hydroxyl moiety of analog **4o** docked at the interior of the *α*-glucosidase pocket and carboxyl moiety positioned at the entrance of the pocket (Figures 4A,B). Detailed binding results (Figures 4C,D) pointed that analog **4o** formed a hydrogen bond with Asp 300 (2.0 Å), a π–π stacking with Trp 59 (4.6 Å), and hydrophobic interactions with Leu 162 and His 305.

### Cell Cytotoxicity Assay

**FIGURE 5:**
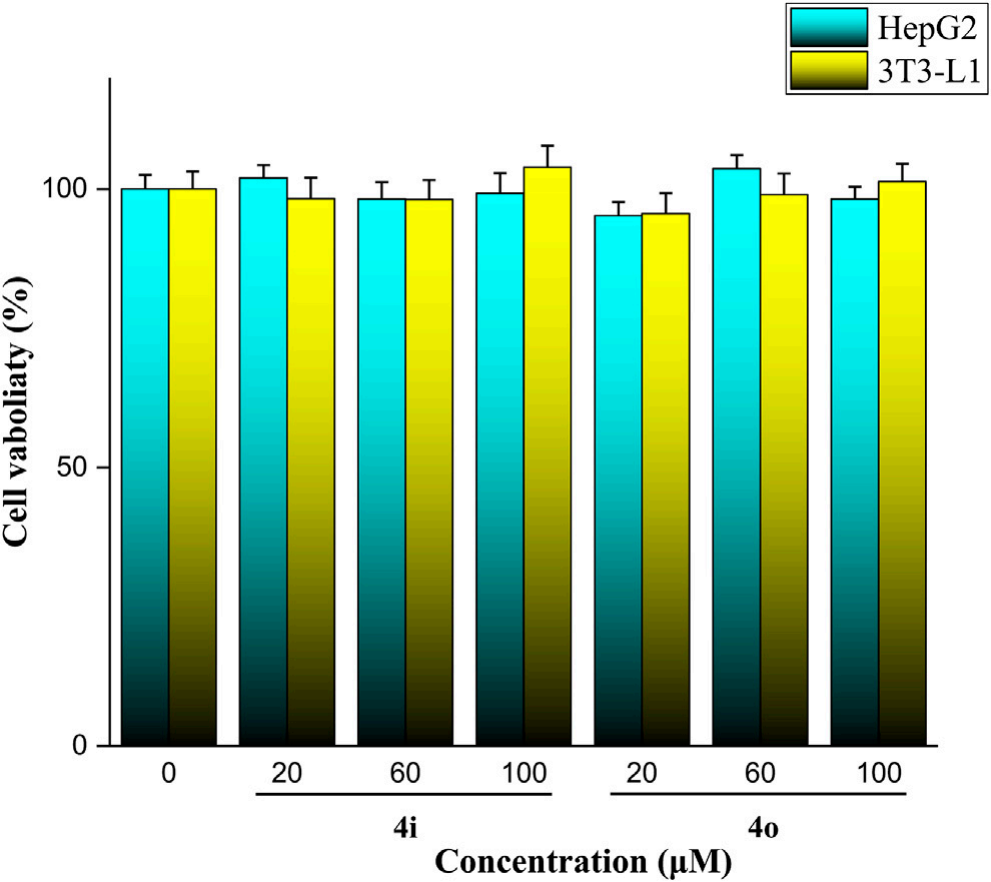
Cell cytotoxicity of analogs **4i** and **4o** against HepG2 and 3T3-L1 cells.

The cell cytotoxicity of strongest inhibitory activity analogs **4i** and **4o** was evaluated against HepG2 and 3T3-L1 cells. The results showed that analogs **4i** and **4o** had a non-cytotoxic effect to HepG2 and 3T3-L1 cells under a concentration of 100 μM (Figure 5).

## Conclusion

In summary, benzylidene analogs of oleanolic acid **4a**–**4s** were synthesized for finding potential *α*-glucosidase and *α*-amylase inhibitors. All synthesized analogs displayed bifunctionality against *α*-glucosidase and *α*-amylase. Analog **4i** showed the highest *α*-glucosidase inhibition (IC_50_: 0.40 μM), and analog **4o** presented the strongest *α*-amylase inhibition (IC_50_: 9.59 μM). Inhibition kinetics results showed that analogs **4i** and **4o** were reversible and had mixed-type inhibitors against *α*-glucosidase and *α*-amylase, respectively. Moreover, analogs **4i** and **4o** showed a high level of safety against HepG2 and 3T3-L1 cells, which provided strong support for the further *in vivo* assay. In viewpoint of all these experimental data, benzylidene analogs of oleanolic acid have the potential to be developed into the leading compound in the management of T2D.

## Experimental

### Materials and Methods

OA was purchased from Energy Chemical Company, China. *α*-glucosidase from *Saccharomyces cerevisiae* (EC 3.2.1.20), *α*-amylase from hog pancreas (EC 3.2.1.1), and *p*-nitrophenyl-α-D-galactopyranoside (*p*-NPG) were provided by Sigma-Aldrich. Water-soluble starch was obtained from Shanghai Yuanye Biological Technology Co., Ltd. 3T3-L1 cells and HepG2 cells were supplied by ATCC. Other reagents were purchased from commercial suppliers. All the compounds were dissolved in DMSO, and DMSO working concentration in the enzyme inhibition test was 5%. The NMR spectra were recorded on a Bruker AM spectrometer (500 MHz). High-resolution mass spectral analysis (HRMS) was carried out on the Apex II by means of the ESI technique. Melting points were detected on a WRS-2C micro melting point instrument.

### Synthesis of OA Analogs 4a–4s

To a solution of oleanolic acid (1.0 mmol) in acetone (10 ml) was added freshly prepared Jones reagent at 0°C for 1 h. The mixture was quenched with methanol, then extracted with ethyl acetate, dried over Na_2_SO_4_, and concentrated *in vacuo*. Then, the crude product was purified by silica gel column chromatography to yield compound **2**. Then, compound **2** (0.5 mmol) was condensed with substituted aromatic aldehydes (0.75 mmol) at room temperature overnight. After the completion of the reaction, the mixture was adjusted to pH = 1 with 1N diluted hydrochloric acid, then extracted with ethyl acetate, washed with water, dried by Na_2_SO_4,_ and evaporated to dryness. Compounds **3a**–**3s** were obtained through the purification of silica gel column chromatography.

Last, NaBH_4_ (1.25 mmol) was added to the solution of **3a**–**3s** (0.25 mmol) in methanol (2 ml) at 0 C. The mixture was warmed to room temperature and stirred for 1–2 h. The mixture was quenched with cold water, extracted with ethyl acetate, dried by Na_2_SO_4,_ and separated by silica gel column chromatography to the desired product **4a**–**4s**.

***(4a, C***_***37***_ ***H***_***52***_ ***O***_***3***_ ***).*** White solid; Yield: 75%; mp: 183–184 °C; ^1^H NMR (500 MHz, chloroform-*d*) δ 7.34–7.29 (m, 2H), 7.22 (d, *J* = 7.8 Hz, 3H), 6.69 (s, 1H), 5.22 (t, *J* = 3.6 Hz, 1H), 3.87 (d, *J* = 2.0 Hz, 1H), 2.92 (d, *J* = 12.9 Hz, 1H), 2.79 (dd, *J* = 13.8, 4.5 Hz, 1H), 1.96 (td, *J* = 13.5, 4.1 Hz, 1H), 1.82–1.52 (m, 9H), 1.47 (td, *J* = 12.4, 3.8 Hz, 1H), 1.43–1.17 (m, 5H), 1.14 (s, 4H), 1.13 (s, 4H), 1.11–1.03 (m, 2H), 0.91 (s, 3H), 0.89 (s, 3H), 0.78 (s, 3H), 0.75 (s, 3H), and 0.68 (s, 3H). ^13^C NMR (126 MHz, CDCl_3_) δ 184.19, 143.63, 140.33, 138.13, 128.96, 128.26, 126.20, 122.67, 122.56, 81.26, 55.90, 47.02, 46.65, 45.95, 41.78, 41.74, 41.69, 41.07, 40.36, 39.57, 33.88, 33.21, 32.58, 32.53, 30.80, 28.76, 27.79, 26.04, 23.71, 23.46, 22.95, 18.51, 17.12, 15.78, and 15.61. HRMS (ESI-MS) m/z: [M + Na]^+^ calculated for C_37_H_52_O_3_Na: 567.3809; found: 567.3809.

***(4b, C***_***38***_ ***H***_***54***_ ***O***_***3***_ ***)***. White solid; Yield: 65%; mp: 210–211 °C; ^1^H NMR (500 MHz, chloroform-*d*) δ 7.15 (ddt, *J* = 17.2, 5.9, 3.3 Hz, 3H), 7.04 (dd, *J* = 6.5, 2.3 Hz, 1H), 6.64 (s, 1H), 5.16 (t, *J* = 3.2 Hz, 1H), 3.92 (d, *J* = 1.9 Hz, 1H), 2.76 (dd, *J* = 13.8, 4.5 Hz, 1H), 2.60 (d, *J* = 12.8 Hz, 1H), 2.24 (s, 3H), 1.93 (td, *J* = 13.4, 4.0 Hz, 1H), 1.78–1.49 (m, 8H), 1.49–1.23 (m, 6H), 1.21–1.00 (m, 10H), 0.89 (d, *J* = 3.2 Hz, 7H), 0.78 (s, 3H), 0.66 (s, 3H), and 0.63 (s, 3H). ^13^C NMR (126 MHz, CDCl_3_) δ 184.20, 143.55, 139.85, 137.27, 136.55, 129.84, 129.42, 126.53, 125.42, 122.56, 122.12, 81.17, 55.76, 47.09, 46.64, 45.94, 42.14, 41.70, 41.47, 41.08, 40.07, 39.54, 33.86, 33.21, 32.56, 30.78, 28.68, 27.74, 26.05, 23.69, 23.44, 22.90, 20.20, 18.55, 17.04, 15.80, and 15.32. HRMS (ESI-MS) m/z: [M + H]^+^ calculated for C_38_H_55_O_3_: 559.4146; found: 559.4148.

***(4c, C***_***38***_ ***H***_***54***_ ***O***_***3***_ ***)*.** White solid; Yield: 71%; mp: 128–129 °C; ^1^H NMR (500 MHz, chloroform-*d*) δ 7.20 (t, *J* = 7.5 Hz, 1H), 7.06–7.00 (m, 3H), 6.66 (s, 1H), 5.23 (t, *J* = 3.6 Hz, 1H), 3.86 (d, *J* = 2.1 Hz, 1H), 2.94 (d, *J* = 12.9 Hz, 1H), 2.80 (dd, *J* = 13.9, 4.6 Hz, 1H), 2.34 (s, 3H), 1.96 (td, *J* = 13.5, 4.1 Hz, 1H), 1.87–1.52 (m, 9H), 1.52–1.18 (m, 6H), 1.17–1.01 (m, 9H), 0.90 (d, *J* = 7.9 Hz, 7H), 0.80 (s, 3H), 0.74 (s, 3H), and 0.69 (s, 3H). ^13^C NMR (126 MHz, CDCl_3_) δ 184.27, 143.68, 140.10, 138.03, 137.70, 129.76, 128.13, 126.95, 125.96, 122.68, 122.58, 81.29, 55.93, 47.00, 46.66, 45.96, 41.81, 41.75, 41.67, 41.06, 40.35, 39.57, 33.89, 33.20, 32.58, 32.53, 30.79, 28.75, 27.81, 26.04, 23.70, 23.43, 22.96, 21.64, 18.50, 17.14, 15.78, and 15.67. HRMS (ESI-MS) m/z: [M + Na]^+^ calculated for C_38_H_54_O_3_Na: 581.3965; found: 581.3959.

***(4d, C***_***38***_ ***H***_***54***_ ***O***_***3***_ ***)*.** White solid; Yield: 73%; mp: 146–147 °C; ^1^H NMR (500 MHz, chloroform-*d*) δ 7.12 (s, 4H), 6.65 (s, 1H), 5.22 (d, *J* = 3.7 Hz, 1H), 3.86 (d, *J* = 2.0 Hz, 1H), 2.94 (d, *J* = 12.9 Hz, 1H), 2.79 (dd, *J* = 13.8, 4.6 Hz, 1H), 2.35 (s, 3H), 1.96 (td, *J* = 13.5, 4.1 Hz, 1H), 1.86–1.51 (m, 9H), 1.51–1.43 (m, 1H), 1.38 (t, *J* = 8.2 Hz, 2H), 1.34–1.26 (m, 4H), 1.23–1.18 (m, 1H), 1.13 (d, *J* = 1.7 Hz, 6H), 1.05 (td, *J* = 13.1, 12.5, 2.8 Hz, 2H), 0.91 (s, 3H), 0.89 (s, 3H), 0.78 (s, 3H), 0.74 (s, 3H), and 0.69 (s, 3H). ^13^C NMR (126 MHz, CDCl_3_) δ 184.10, 143.62, 139.69, 135.75, 135.10, 128.99, 128.84, 122.58, 122.47, 81.31, 55.92, 47.02, 46.65, 45.95, 41.79, 41.75, 41.66, 41.09, 40.36, 39.57, 33.89, 33.21, 32.59, 32.53, 30.80, 29.84, 28.74, 27.80, 26.04, 23.71, 23.49, 22.96, 21.30, 18.51, 17.11, 15.77, and 15.61. HRMS (ESI-MS) m/z: [M + H]^+^ calculated for C_38_H_55_O_3_: 559.4146; found: 559.4149.

***(4e, C***_***38***_ ***H***_***54***_ ***O***_***4***_ ***)*.** White solid; Yield: 72%; mp: 200–201°C; ^1^H NMR (500 MHz, chloroform-*d*) δ 7.22 (td, *J* = 7.9, 1.7 Hz, 1H), 7.14 (dd, *J* = 7.6, 1.6 Hz, 1H), 6.90 (td, *J* = 7.5, 1.1 Hz, 1H), 6.87 (dd, *J* = 8.2, 1.0 Hz, 1H), 6.65 (s, 1H), 5.19 (t, *J* = 3.7 Hz, 1H), 3.89 (d, J = 2.0 Hz, 1H), 3.80 (s, 3H), 2.77 (dd, *J* = 13.8, 4.6 Hz, 1H), 2.72 (d, *J* = 12.9 Hz, 1H), 1.95 (td, *J* = 13.5, 4.0 Hz, 1H), 1.78–1.69 (m, 3H), 1.68–1.52 (m, 5H), 1.49–1.23 (m, 5H), 1.22–1.00 (m, 10H), 0.89 (d, *J* = 7.0 Hz, 7H), 0.76 (d, *J* = 9.7 Hz, 6H), and 0.67 (s, 3H). ^13^C NMR (126 MHz, CDCl_3_) δ 184.04, 157.38, 143.57, 140.11, 130.12, 127.72, 127.04, 122.63, 120.16, 118.57, 110.58, 81.39, 55.91, 55.55, 47.05, 46.65, 45.95, 42.21, 41.75, 41.58, 41.08, 40.18, 39.56, 33.88, 33.20, 32.62, 32.52, 30.79, 28.71, 27.79, 26.04, 23.71, 23.46, 22.96, 18.51, 17.12, 15.80, and 15.36. HRMS (ESI-MS) m/z: [M + Na]^+^ calculated for C_38_H_54_O_4_Na: 597.3914; found: 597.3925.

***(4f, C***_***38***_ ***H***_***54***_ ***O***_***4***_ ***)*.** White solid; Yield: 62%; mp: 200–201°C; ^1^H NMR (500 MHz, chloroform-*d*) δ 7.15 (d, *J* = 8.7 Hz, 2H), 6.86 (d, *J* = 8.7 Hz, 2H), 6.62 (s, 1H), 5.23 (t, *J* = 3.6 Hz, 1H), 3.85 (d, *J* = 2.0 Hz, 1H), 3.82 (s, 3H), 2.92 (d, *J* = 12.9 Hz, 1H), 2.79 (dd, *J* = 13.9, 4.6 Hz, 1H), 1.96 (td, *J* = 13.5, 4.1 Hz, 1H), 1.86–1.70 (m, 2H), 1.70–1.52 (m, 6H), 1.47 (td, *J* = 12.3, 4.0 Hz, 1H), 1.40–1.32 (m, 3H), 1.31–1.24 (m, 4H), 1.23–1.18 (m, 1H), 1.13 (d, *J* = 3.2 Hz, 6H), 1.10–1.01 (m, 2H), 0.91 (s, 3H), 0.89 (s, 3H), 0.78 (s, 3H), 0.73 (s, 3H), and 0.69 (s, 3H). ^13^C NMR (126 MHz, CDCl_3_) δ 184.06, 159.49, 143.69, 140.54, 139.51, 129.23, 122.56, 122.53, 121.55, 114.09, 112.13, 81.27, 55.94, 55.34, 47.01, 46.65, 45.96, 41.87, 41.76, 41.72, 41.06, 40.36, 39.57, 33.89, 33.20, 32.59, 32.52, 30.80, 28.76, 27.81, 26.04, 23.70, 23.50, 22.98, 18.50, 17.14, 15.80, and 15.70. HRMS (ESI-MS) m/z: [M + Na]^+^ calculated for C_38_H_54_O_4_Na: 597.3914; found: 597.3915.

***(4g, C***_***38***_ ***H***_***54***_ ***O***_***4***_ ***)*.** White solid; Yield: 70%; mp: 296–297°C; ^1^H NMR (500 MHz, chloroform-*d*) δ 7.15 (d, *J* = 8.7 Hz, 2H), 6.86 (d, *J* = 8.7 Hz, 2H), 6.62 (s, 1H), 5.23 (t, *J* = 3.6 Hz, 1H), 3.85 (d, *J* = 2.0 Hz, 1H), 3.82 (s, 3H), 2.92 (d, *J* = 12.9 Hz, 1H), 2.79 (dd, *J* = 13.9, 4.6 Hz, 1H), 1.96 (td, *J* = 13.5, 4.1 Hz, 1H), 1.86–1.70 (m, 2H), 1.70–1.52 (m, 6H), 1.47 (td, *J* = 12.3, 4.0 Hz, 1H), 1.40–1.32 (m, 3H), 1.31–1.24 (m, 4H), 1.23–1.18 (m, 1H), 1.13 (d, *J* = 3.2 Hz, 6H), 1.10–1.01 (m, 2H), 0.91 (s, 3H), 0.89 (s, 3H), 0.78 (s, 3H), 0.73 (s, 3H), and 0.69 (s, 3H). ^13^C NMR (126 MHz, CDCl3) δ 184.16, 157.96, 143.64, 139.12, 130.56, 130.05, 122.57, 122.03, 113.69, 81.31, 55.91, 55.37, 47.00, 46.65, 45.95, 41.75, 41.62, 41.08, 40.32, 39.57, 33.88, 33.21, 32.59, 32.53, 30.80, 29.84, 28.73, 27.79, 26.04, 23.71, 23.50, 22.96, 18.50, 17.11, 15.77, and 15.60. HRMS (ESI-MS) m/z: [M + H]^+^ calculated for C_38_H_55_O_4_: 575.4095; found: 575.4072.

***(4h, C***_***37***_ ***H***_***51***_ ***FO***_***3***_ ***)*.** White solid; Yield: 75%; mp: 165–166 °C; ^1^H NMR (500 MHz, chloroform-*d*) δ 7.26–7.17 (m, 2H), 7.13–7.03 (m, 2H), 6.63 (s, 1H), 5.21 (t, J = 3.6 Hz, 1H), 3.93 (d, J = 1.9 Hz, 1H), 2.79 (dd, J = 13.9, 4.6 Hz, 1H), 2.65 (d, J = 12.9 Hz, 1H), 1.97 (td, J = 13.5, 4.1 Hz, 1H), 1.75 (qd, J = 9.1, 8.5, 5.8 Hz, 3H), 1.70–1.53 (m, 5H), 1.53–1.43 (m, 2H), 1.41–1.19 (m, 7H), 1.19–1.04 (m, 8H), 0.92 (s, 3H), 0.91 (s, 3H), 0.79 (s, 3H), 0.73 (s, 3H), and 0.68 (s, 3H). ^13^C NMR (126 MHz, CDCl_3_) δ184.15, 161.29, 159.34, 143.16, 130.85, 128.14, 125.80, 123.67, 122.49, 115.88, 115.46, 81.20, 55.78, 47.05, 46.64, 45.94, 42.37, 41.73, 41.69, 41.06, 40.21, 39.55, 33.86, 33.20, 32.53, 30.78, 29.85, 28.72, 27.76, 26.04, 23.70, 23.41, 22.94, 18.54, 17.05, 15.74, and 15.32.HRMS (ESI-MS) m/z: [M + H]^+^ calculated for C_37_H_52_FO_3_: 563.3895; found: 563.3904.

***(4i, C***_***37***_ ***H***_***51***_ ***FO***_***3***_ ***)*.** White solid; Yield: 69%; mp: 157–158 °C; ^1^H NMR (500 MHz, chloroform-*d*) δ 7.28 (d, J = 7.9 Hz, 1H), 6.97 (d, J = 7.7 Hz, 1H), 6.91 (d, J = 9.4 Hz, 2H), 6.66 (s, 1H), 5.25–5.18 (m, 1H), 3.87 (d, J = 2.0 Hz, 1H), 2.89–2.75 (m, 2H), 1.95 (td, J = 13.5, 4.0 Hz, 1H), 1.83–1.51 (m, 8H), 1.51–1.17 (m, 9H), 1.13 (d, J = 2.6 Hz, 7H), 0.90 (d, J = 8.2 Hz, 7H), 0.76 (s, 3H), 0.74 (s, 3H), and 0.68 (s, 3H). ^13^C NMR (126 MHz, CDCl_3_) δ 184.38, 163.77, 161.83, 143.68, 141.52, 140.47, 129.62, 124.69, 122.11, 115.66, 113.04, 81.14, 55.82, 47.00, 46.64, 45.94, 41.77, 41.75, 41.72, 41.05, 40.40, 39.55, 33.87, 33.19, 32.52, 30.78, 29.83, 28.74, 27.77, 26.02, 23.69, 23.41, 22.92, 18.49, 17.09, 15.76, and 15.59. HRMS (ESI-MS) m/z: [M + H]^+^ calculated for C_37_H_52_FO_3_: 563.3895; found: 563.3879.

***(4j, C***_***37***_ ***H***_***51***_ ***FO***_***3***_ ***).*** White solid; Yield: 57%; mp: 226–227°C; ^1^H NMR (500 MHz, chloroform-*d*) δ 7.18–7.13 (m, 2H), 7.00 (t, J = 8.5 Hz, 2H), 6.65 (s, 1H), 5.24–5.20 (m, 1H), 3.86 (d, J = 1.9 Hz, 1H), 2.80 (dd, J = 16.4, 11.2 Hz, 2H), 1.95 (td, J = 13.5, 4.1 Hz, 1H), 1.85–1.51 (m, 10H), 1.50–1.16 (m, 7H), 1.13 (s, 6H), 1.10–1.01 (m, 2H), 0.91 (s, 3H), 0.89 (s, 3H), 0.75 (s, 3H), 0.73 (s, 3H), and 0.68 (s, 3H). ^13^C NMR (126 MHz, CDCl_3_) δ 184.15, 161.38, 143.69, 140.42, 134.08, 130.41, 122.47, 121.69, 115.14, 81.17, 55.83, 47.01, 46.64, 45.95, 41.75, 41.69, 41.67, 41.08, 40.35, 39.57, 33.88, 33.20, 32.56, 32.53, 30.80, 28.75, 27.78, 26.04, 23.71, 23.45, 22.94, 18.50, 17.10, 15.77, and 15.57. HRMS (ESI-MS) m/z: [M + Na]^+^ calculated for C_37_H_52_FO_3_: 563.3895; found: 563.3899.

***(4k, C***_***37***_ ***H***_***51***_ ***ClO***_***3***_ ***).*** White solid; Yield: 58%; mp: 219–220 °C; ^1^H NMR (500 MHz, chloroform-*d*) δ7.38 (dd, J = 7.3, 1.8 Hz, 1H), 7.20–7.13 (m, 3H), 6.67 (s, 1H), 5.17 (d, J = 3.1 Hz, 1H), 3.92 (d, J = 1.9 Hz, 1H), 2.76 (dd, J = 14.0, 4.5 Hz, 1H), 2.58 (d, J = 12.8 Hz, 1H), 1.94 (td, J = 13.5, 4.1 Hz, 1H), 1.78–1.50 (m, 10H), 1.44 (td, J = 14.2, 12.6, 9.8 Hz, 2H), 1.38–1.27 (m, 3H), 1.22–1.17 (m, 1H), 1.14 (d, J = 14.9 Hz, 6H), 1.09–1.02 (m, 3H), 0.89 (s, 3H), 0.89 (s, 3H), 0.80 (s, 3H), 0.68 (s, 3H), and 0.64 (s, 3H). ^13^C NMR (126 MHz, CDCl_3_) δ 184.20, 143.64, 141.64, 136.67, 134.09, 130.93, 129.40, 127.79, 126.31, 122.50, 120.78, 81.16, 55.82, 47.09, 46.64, 45.95, 42.22, 41.77, 41.72, 41.08, 40.28, 39.56, 33.86, 33.21, 32.57, 32.52, 30.79, 28.69, 27.76, 26.05, 23.70, 23.42, 22.92, 18.55, 17.07, 15.72, and 15.18. HRMS (ESI-MS) m/z: [M + H]^+^ calculated for C_37_H_52_ClO_3_: 579.3599; found: 579.3577.

***(4l, C***_***37***_ ***H***_***51***_ ***ClO***_***3***_ ***).*** White solid; Yield: 69%; mp: 242–243 °C; ^1^H NMR (500 MHz, chloroform-*d*) δ 7.27–7.15 (m, 3H), 7.08 (d, *J* = 7.5 Hz, 1H), 6.64 (s, 1H), 5.24 (d, *J* = 3.6 Hz, 1H), 3.87 (d, *J* = 2.0 Hz, 1H), 2.81 (dd, *J* = 19.1, 11.1 Hz, 2H), 1.96 (td, *J* = 13.5, 4.1 Hz, 1H), 1.83–1.53 (m, 8H), 1.51–1.40 (m, 2H), 1.41–1.26 (m, 4H), 1.29–1.15 (m, 2H), 1.13 (d, *J* = 3.2 Hz, 7H), 1.10–1.01 (m, 2H), 0.90 (d, *J* = 8.1 Hz, 6H), 0.75 (d, *J* = 12.9 Hz, 6H), and 0.68 (s, 3H). ^13^C NMR (126 MHz, CDCl_3_) δ 184.11, 143.69, 141.76, 140.05, 134.06, 129.49, 128.97, 127.12, 126.29, 122.50, 121.60, 81.14, 55.83, 47.01, 46.64, 45.95, 41.79, 41.77, 41.75, 41.06, 40.45, 39.57, 33.88, 33.20, 32.53, 30.80, 28.77, 27.79, 26.04, 23.70, 23.41, 22.94, 18.51, 17.12, 15.79, and 15.65. HRMS (ESI-MS) m/z: [M + H]^+^ calculated for C_37_H_52_ClO_3_: 579.3599; found: 579.3572.

***(4m, C***_***37***_ ***H***_***51***_ ***ClO***_***3***_ ***).*** White solid; Yield: 57%; mp: 196–197 °C; ^1^H NMR (500 MHz, chloroform-*d*) δ 7.28 (d, *J* = 8.4 Hz, 2H), 7.13 (d, *J* = 8.3 Hz, 2H), 6.64 (s, 1H), 5.23 (t, *J* = 3.7 Hz, 1H), 3.86 (d, *J* = 2.1 Hz, 1H), 2.81 (t, *J* = 13.1 Hz, 2H), 1.96 (td, *J* = 13.5, 4.1 Hz, 1H), 1.82–1.52 (m, 8H), 1.55–1.17 (m, 8H), 1.13 (s, 7H), 1.06 (ddd, *J* = 17.3, 10.9, 4.1 Hz, 2H), 0.90 (d, *J* = 10.2 Hz, 6H), 0.74 (d, J = 7.3 Hz, 6H), and 0.68 (s, 3H). ^13^C NMR (126 MHz, CDCl_3_) δ 184.13, 143.70, 141.12, 136.60, 131.86, 130.26, 128.42, 122.42, 121.66, 81.16, 55.81, 47.02, 46.63, 45.94, 41.74, 41.72, 41.06, 40.41, 39.57, 33.87, 33.20, 32.54, 32.52, 30.79, 28.75, 27.77, 26.04, 23.71, 23.48, 22.93, 18.50, 17.08, 15.78, and 15.58. HRMS (ESI-MS) m/z: [M + K]^+^ calculated for C_37_H_51_ClO_3_K: 617.3158; found: 617.3146.

***(4n, C***_***37***_ ***H***_***51***_ ***BrO***_***3***_ ***).*** White solid; Yield: 58%; mp: 234–235 °C; ^1^H NMR (500 MHz, chloroform-*d*) δ 7.57 (d, J = 7.9 Hz, 1H), 7.24 (d, J = 7.6 Hz, 1H), 7.13 (d, J = 7.6 Hz, 1H), 7.09 (t, J = 7.8 Hz, 1H), 6.62 (s, 1H), 5.17 (d, J = 3.0 Hz, 1H), 3.91 (d, J = 1.9 Hz, 1H), 2.76 (dd, J = 13.9, 4.5 Hz, 1H), 2.57 (d, J = 12.8 Hz, 1H), 1.94 (td, J = 13.6, 4.1 Hz, 1H), 1.78–1.61 (m, 5H), 1.55 (dd, J = 22.2, 13.7 Hz, 4H), 1.49–1.23 (m, 5H), 1.08 (dd, J = 50.0, 12.8 Hz, 11H), 0.89 (d, J = 4.4 Hz, 6H), 0.81 (s, 3H), 0.69 (s, 3H), and 0.64 (s, 3H). ^13^C NMR (126 MHz, CDCl_3_) δ 183.95, 143.63, 141.30, 138.47, 132.57, 131.01, 128.00, 126.95, 124.65, 123.04, 122.51, 81.11, 55.81, 47.10, 46.63, 45.94, 42.17, 41.84, 41.72, 41.10, 40.32, 39.56, 33.86, 33.21, 32.58, 32.52, 30.79, 28.70, 27.75, 26.04, 23.70, 23.42, 22.92, 18.55, 17.08, 15.81, and 15.21. HRMS (ESI-MS) m/z: [M + H]^+^ calculated for C_37_H_52_BrO_3_: 623.3094; found: 623.3067.

***(4o, C***_***37***_ ***H***_***51***_ ***BrO***_***3***_ ***).*** White solid; Yield: 60%; mp: 197–198 °C; ^1^H NMR (500 MHz, chloroform-*d*) δ 7.38–7.31 (m, 2H), 7.17 (t, *J* = 7.8 Hz, 1H), 7.12 (d, *J* = 7.7 Hz, 1H), 6.64 (s, 1H), 5.26–5.21 (m, 1H), 3.87 (d, *J* = 1.9 Hz, 1H), 2.88–2.74 (m, 2H), 1.95 (td, *J* = 13.5, 4.1 Hz, 1H), 1.83–1.51 (m, 8H), 1.51–1.24 (m, 7H), 1.23–1.17 (m, 1H), 1.16–1.01 (m, 9H), 0.90 (d, *J* = 8.0 Hz, 6H), 0.75 (d, *J* = 13.9 Hz, 6H), and 0.68 (s, 3H). ^13^C NMR (126 MHz, CDCl_3_) δ 184.31, 143.69, 141.80, 140.35, 131.86, 129.77, 129.17, 127.55, 122.49, 122.33, 121.50, 81.14, 55.84, 47.01, 46.64, 45.95, 41.79, 41.74, 41.04, 40.46, 39.57, 33.88, 33.20, 32.53, 30.79, 29.83, 28.77, 27.79, 23.70, 23.39, 22.93, 18.50, 17.12, 15.79, and 15.65. HRMS (ESI-MS) m/z: [M + H]^+^ calculated for C_37_H_52_BrO_3_: 623.3094; found: 623.3087.

***(4p, C***_***37***_ ***H***_***51***_ ***BrO***_***3***_ ***).*** White solid; Yield: 66%; mp: 224–225 °C; ^1^H NMR (500 MHz, chloroform-d) δ7.43 (d, J = 8.3 Hz, 2H), 7.07 (d, J = 8.2 Hz, 2H), 6.62 (s, 1H), 5.34–5.09 (m, 1H), 3.86 (d, J = 2.0 Hz, 1H), 2.81 (t, J = 12.5 Hz, 2H), 1.96 (td, J = 13.5, 4.1 Hz, 1H), 1.85–1.51 (m, 7H), 1.51–1.17 (m, 9H), 1.13 (s, 9H), 0.91 (s, 3H), 0.89 (s, 3H), 0.74 (s, 3H), 0.73 (s, 3H), and 0.68 (s, 3H). ^13^C NMR (126 MHz, CDCl_3_) δ 184.11, 143.69, 141.22, 137.09, 131.38, 130.63, 122.44, 121.70, 120.01, 81.18, 55.82, 47.04, 46.64, 45.95, 41.75, 41.73, 41.08, 40.43, 39.58, 33.88, 33.21, 32.55, 32.53, 30.80, 29.85, 28.76, 27.78, 26.05, 23.72, 23.50, 22.94, 18.52, 17.09, 15.79, and 15.60. HRMS (ESI-MS) m/z: [M + H]^+^ calculated for C_37_H_52_BrO_3_: 623.3094; found: 623.3067.

***(4q, C***_***37***_ ***H***_***51***_ ***NO***_***5***_ ***)***. White solid; Yield: 55%; mp: 197–198 °C; ^1^H NMR (500 MHz, chloroform-*d*) δ 8.02 (dd, J = 8.2, 1.3 Hz, 1H), 7.54 (td, J = 7.5, 1.4 Hz, 1H), 7.46–7.35 (m, 1H), 7.24 (d, J = 7.7 Hz, 1H), 6.89 (s, 1H), 5.15 (d, J = 2.7 Hz, 1H), 3.90 (d, J = 1.8 Hz, 1H), 2.74 (dd, J = 13.9, 4.5 Hz, 1H), 2.41 (d, J = 12.8 Hz, 1H), 1.94 (td, J = 13.2, 4.1 Hz, 1H), 1.71 (td, J = 13.9, 4.5 Hz, 1H), 1.56 (dt, J = 15.8, 7.4 Hz, 6H), 1.44 (d, J = 13.0 Hz, 2H), 1.37–1.24 (m, 5H), 1.19–1.01 (m, 10H), 0.88 (d, J = 1.8 Hz, 7H), 0.82 (s, 3H), and 0.63 (d, J = 4.2 Hz, 6H). ^13^C NMR (126 MHz, CDCl_3_) δ 183.70, 148.59, 143.64, 141.68, 133.92, 132.66, 132.31, 127.42, 124.63, 122.18, 119.76, 80.98, 55.62, 46.89, 46.50, 45.84, 42.32, 41.81, 41.64, 40.95, 40.09, 39.43, 33.74, 33.07, 32.43, 32.33, 30.66, 28.62, 27.63, 25.89, 23.58, 23.25, 22.85, 18.45, 16.92, 15.56, and 14.94. HRMS (ESI-MS) m/z: [M + K]^+^ calculated for C_37_H_51_NO_5_K: 628.3399; found: 628.3420.

***(4r, C***_***35***_ ***H***_***51***_ ***NO***_***5***_ ***).*** White solid; Yield: 64%; mp: 191–192 °C; ^1^H NMR (500 MHz, chloroform-*d*) δ8.11 (d, J = 2.0 Hz, 1H), 8.07 (dt, J = 7.7, 1.8 Hz, 1H), 7.54–7.46 (m, 2H), 6.76 (s, 1H), 5.22 (d, J = 3.6 Hz, 1H), 3.90 (d, J = 2.0 Hz, 1H), 2.85–2.74 (m, 2H), 1.96 (td, J = 13.5, 4.0 Hz, 1H), 1.82–1.65 (m, 4H), 1.63–1.53 (m, 4H), 1.47 (d, J = 12.6 Hz, 2H), 1.38–1.28 (m, 3H), 1.25 (d, J = 2.9 Hz, 1H), 1.14 (d, J = 9.3 Hz, 7H), 1.11–0.97 (m, 3H), 0.89 (d, J = 7.5 Hz, 7H), 0.77 (d, J = 17.2 Hz, 6H), and 0.69 (s, 3H). ^13^C NMR (126 MHz, CDCl_3_) δ 183.73, 148.21, 143.60, 143.21, 139.79, 135.02, 129.08, 123.51, 122.26, 121.09, 120.74, 80.95, 55.68, 46.92, 46.51, 45.83, 41.84, 41.65, 41.59, 40.94, 40.53, 39.47, 33.76, 33.07, 32.42, 32.39, 30.68, 28.69, 27.67, 25.91, 23.57, 23.27, 22.83, 18.39, 16.99, 15.70, and 15.56. HRMS (ESI-MS) m/z: [M + H]^+^ calculated for C_37_H_51_NO_5_: 590.3840; found: 590.3849.

***(4s, C***_***34***_ ***H***_***51***_ ***NO***_***5***_ ***).*** White solid; Yield: 50%; mp: 219–220 °C; ^1^H NMR (500 MHz, chloroform-*d*) δ 8.19–8.16 (m, 2H), 7.37–7.31 (m, 2H), 6.77 (s, 1H), 5.22 (t, *J* = 3.6 Hz, 1H), 3.91 (d, *J* = 1.9 Hz, 1H), 2.86–2.75 (m, 2H), 1.95 (td, *J* = 13.5, 4.1 Hz, 1H), 1.80–1.40 (m, 12H), 1.39–1.17 (m, 5H), 1.14 (d, *J* = 8.4 Hz, 6H), 1.11–1.00 (m, 2H), 0.90 (d, *J* = 9.0 Hz, 6H), 0.74 (d, *J* = 3.9 Hz, 6H), and 0.67 (s, 3H). ^13^C NMR (126 MHz, CDCl_3_) δ 184.06, 146.10, 145.44, 144.30, 143.81, 129.58, 123.68, 122.18, 121.45, 81.06, 55.71, 47.06, 46.59, 45.92, 42.02, 41.92, 41.74, 41.05, 40.72, 39.59, 33.85, 33.18, 32.50, 30.77, 29.81, 28.78, 27.75, 26.02, 23.68, 23.46, 22.89, 18.51, 17.04, 15.82, and 15.59. HRMS (ESI-MS) m/z: [M + H]^+^ calculated for C_37_H_51_NO_5_: 590.3840; found: 590.3849.

#### *α*-Glucosidase and *α*-Amylase Inhibition Assay

The *α-*glucosidase inhibition assay of analogs **4a**–**4s** was carried out as in previously reported methods, with minor modifications (Xu et al., 2020). Then, 10 μL *α-*glucosidase and 10 μL test sample (dissolved in DMSO) were added into 130 μL PBS (50 mM phosphate saline buffer, pH 6.8) and incubated for 10 min at 37°C. After adding 50 μL pNPG, the absorbance change of the mixture at 405 nm was monitored. Then, the percent inhibition was calculated (Ademiluyi and Oboh, 2013). All experiments were carried out in quadruplicate.

The *α*-amylase inhibitory activity was performed according to methods followed in previous reports, with minor modification (Hameed et al., 2019). Then, 10 μL *α*-amylase (final concentration 0.25 U/mL), 10 μL test compound, and 80 μL phosphate buffer (20 mM, pH 6.9) were mixed and incubated for 10 min at 37°C. Then, 100 μL starch solution (final concentration 0.5%) was added into the mixture and further incubated for 10 min. After 100 μL DNS (containing 1 M potassium sodium tartrate and 48 mM 3,5-dinitrosalicylic acid) was added, the mixture was boiled for 15 min. After diluting using 900 μL distilled water, the absorbance was measured at 540 nm. Then, the percent inhibition was calculated (Aispuro-Perez et al., 2020). All experiments were carried out in quadruplicate.

The inhibition kinetics of **4i** and **4o** against *α-*glucosidase and *α*-amylase, respectively, was investigated using a similar aforementioned inhibition assay method. The enzyme inhibitory kinetics was obtained by the plots of the enzymatic reaction rate *vs*. enzyme concentration with or without the inhibitor, and the substrate inhibitory kinetics was measured using the Lineweaver–Burk plot of the enzymatic reaction rate *vs*. substrate concentration with or without the inhibitor.

### Molecular Docking

Molecular docking was performed using SYBYL software to investigate the interaction between inhibitors and target protein (Zheng P.-F. et al., 2021; Hu, et al., 2021). The crystal structure of *Saccharomyces cerevisiae α*-glucosidase was not resolved so far. The homology model of *α*-glucosidase was constructed using the protocol reported earlier (Wang, et al., 2017; Wang et al., 2017). In brief, the structure of *Saccharomyces cerevisiae* isomaltase (PDB ID: 3AJ7) was selected as a template, the sequence in FASTA format of *α*-glucosidase was obtained from UniProt (access code P53341), and the homology model was prepared using modeler 10.1 software. The quality of the homology model was verified by Ramachandran plot (Yamamoto et al., 2010). Porcine pancreatic *α*-amylase (PDB: 3BAJ) was retrieved from the Protein Data Bank (Rafique et al., 2020). The target protein was optimized by removing water molecules, adding hydrogen atoms, adding charge, and repairing end residues, followed by the generation of an active pocket. The compounds were charged with Gasteiger–Hückle charges and prepared by an energy minimization program. Thus, the docking between the compounds and target protein was operated in the default format, and the results were visualized by PyMol and Discover studio software.

### Statistical Analysis

All data were presented as mean ± SD. One-way ANOVA was performed to evaluate the difference between groups. *p* < 0.05 was considered significant.

## Data Availability

The original contributions presented in the study are included in the article/Supplementary Material, further inquiries can be directed to the corresponding authors.
