# Supplementary material for: Synthesis of Benzylidene Analogs of Oleanolic Acid as Potential α-Glucosidase and α-Amylase Inhibitors
Source: Front Chem. 2022 Jun 8;10:911232. doi: 10.3389/fchem.2022.911232 (PMC9213889; doi:10.3389/fchem.2022.911232)
Supplement: Supplementary file 1 [file DataSheet1.docx]

Supplementary Material

**Synthesis of novel benzylidene analogues of oleanolic acid as potential α-glucosidase and α-amylase Inhibitors**

Jun-Jie Ke, Jing Lin, Xin Zhang, Xiao-Zheng Wu, Ying-Ying Zheng, Chun-Mei Hu, Yu Kang, Kun Zhang, Zhuang Xiong *, Zhi-Qiang Ma *

School of Biotechnology and Health Sciences, Wuyi University, Jiangmen 529000, P. R. China

*Corresponding authors: Zhuang Xiong, E-mail: wyuchemxz@126.com; Tel./fax: +86 750 3299397.

1. **NMR of compounds…………………………………………………………………………..2-20**
2. **ESI-MS of compounds………………………………………………………………………21-30**
3. **HPLC of compounds………………………………………………………………………...30-39**
4. **Inhibition curves of 4i and 4o…………………………………………………………………40**

**Fig.S1** **4a (**^1^H NMR)

**Fig.S2** **4a (**^13^C NMR)


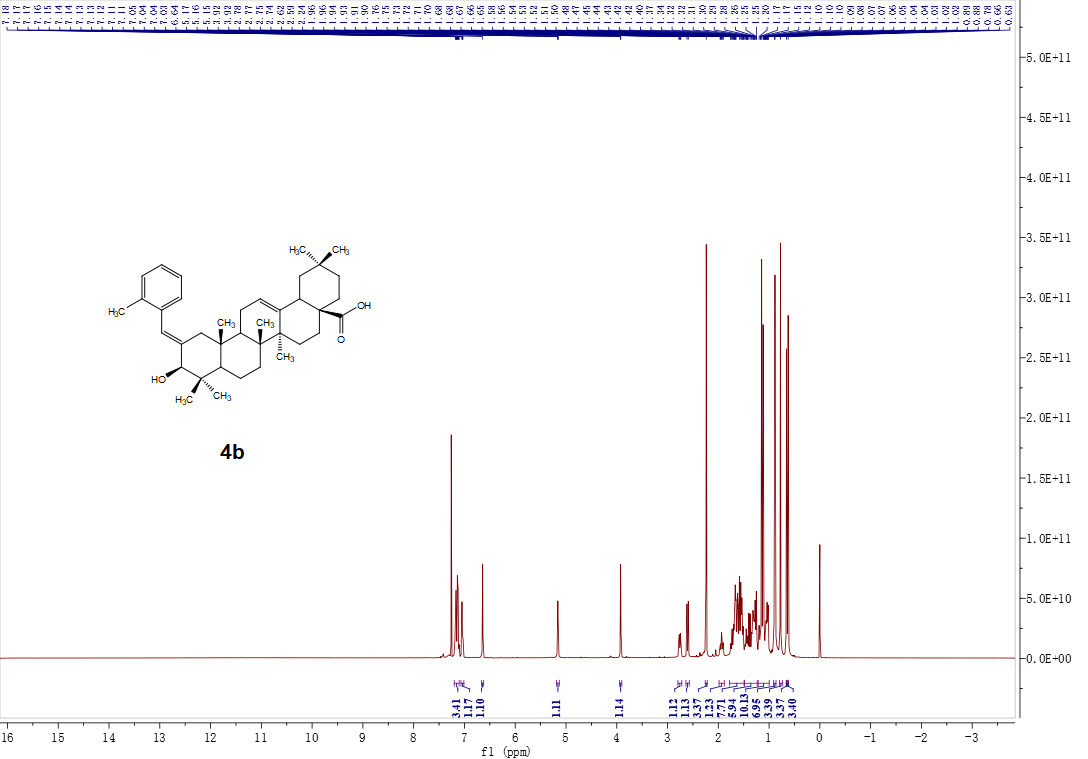


**Fig.S3** 4**b (**^1^H NMR)

**Fig.S4** **4b (**^13^C NMR)


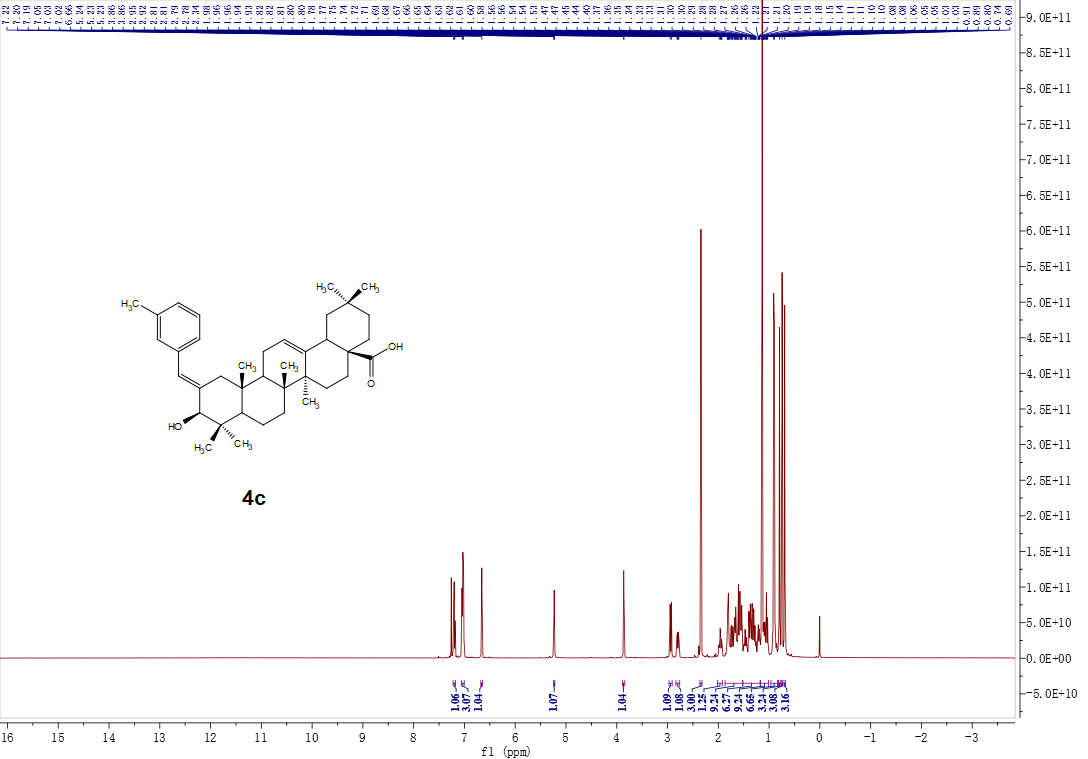


**Fig.S5** 4**c (**^1^H NMR)

**Fig.S6** **4c (**^13^C NMR)

**Fig.S7** 4**d (**^1^H NMR)


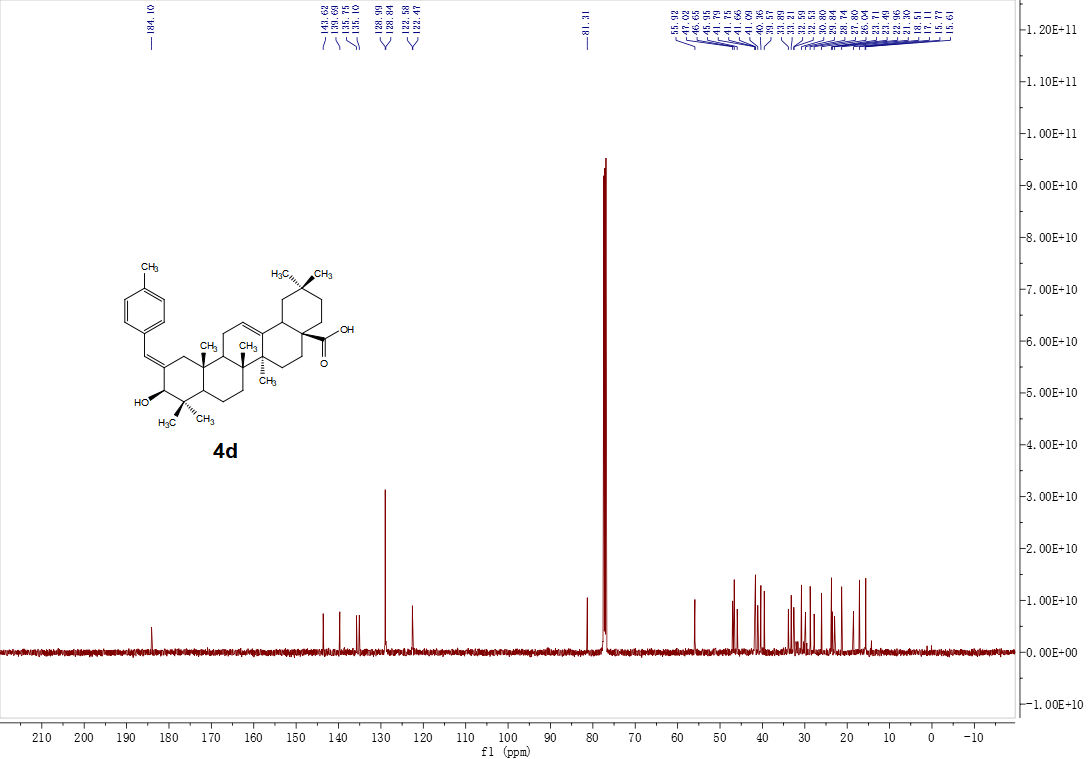


**Fig.S8** 4d **(**^13^C NMR)


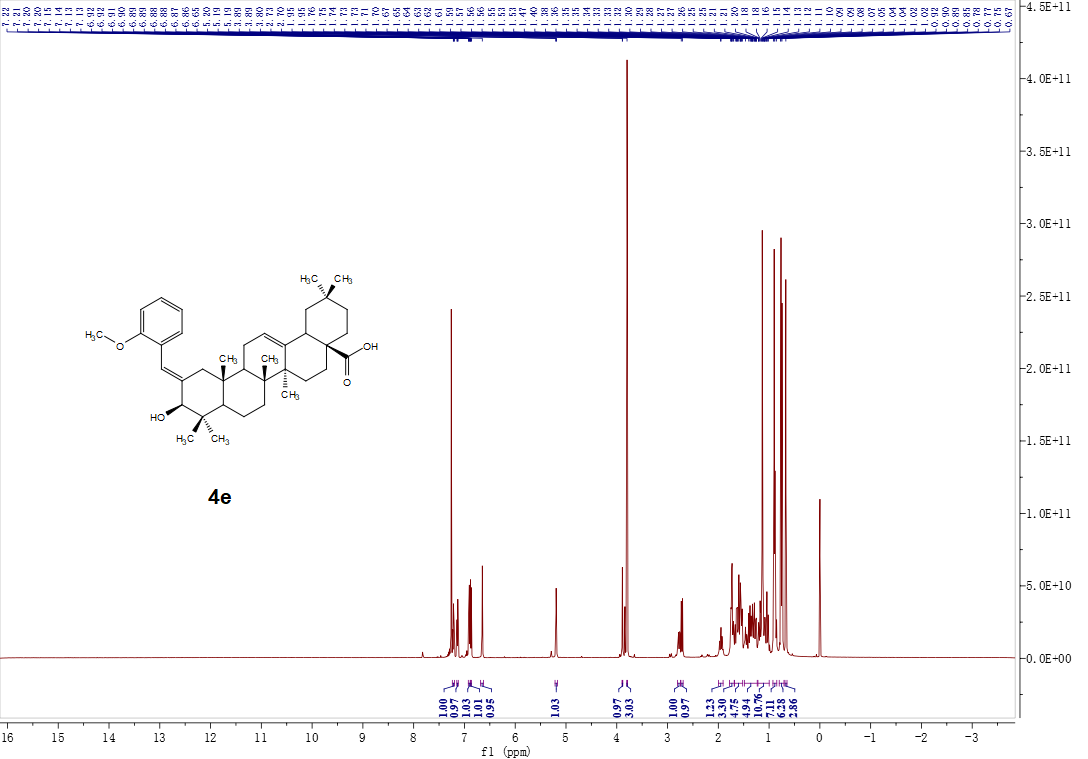


**Fig.S9** 4**e (**^1^H NMR)

**Fig.S10** 4**e (**^13^C NMR)

**Fig.S11** 4f **(**^1^H NMR)

**Fig.S12** 4f **(**^13^C NMR)


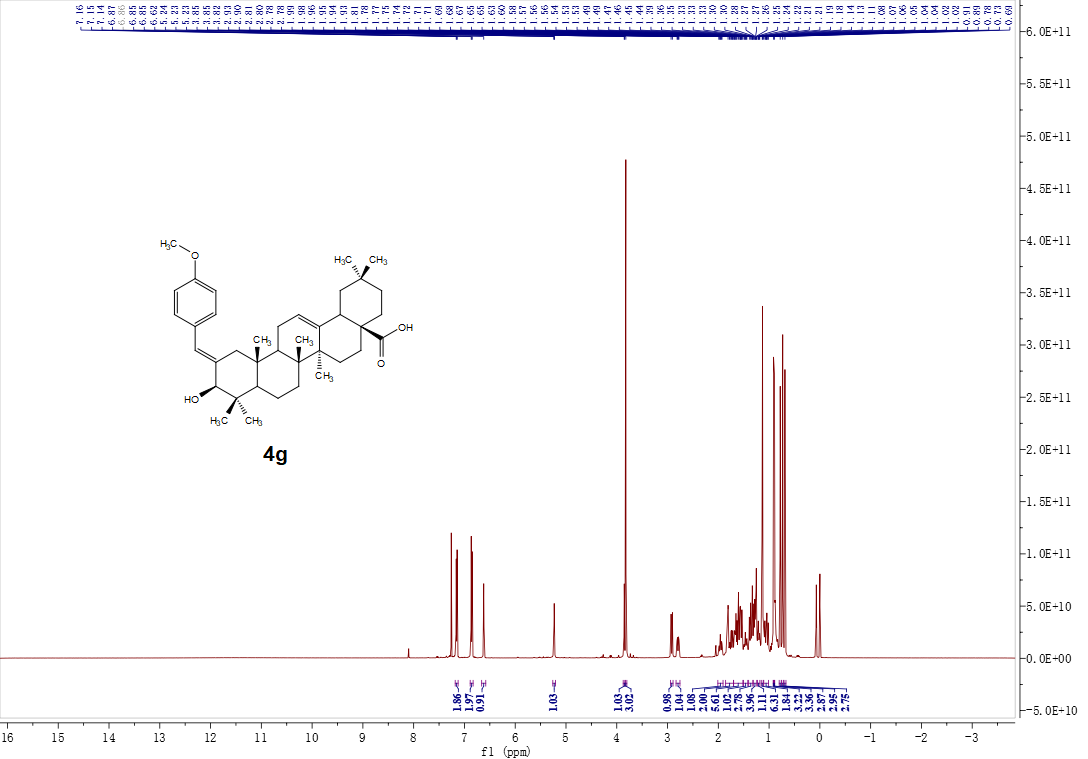


**Fig.S13** 4g **(**^1^H NMR)

**Fig.S14** 4g **(**^13^C NMR)


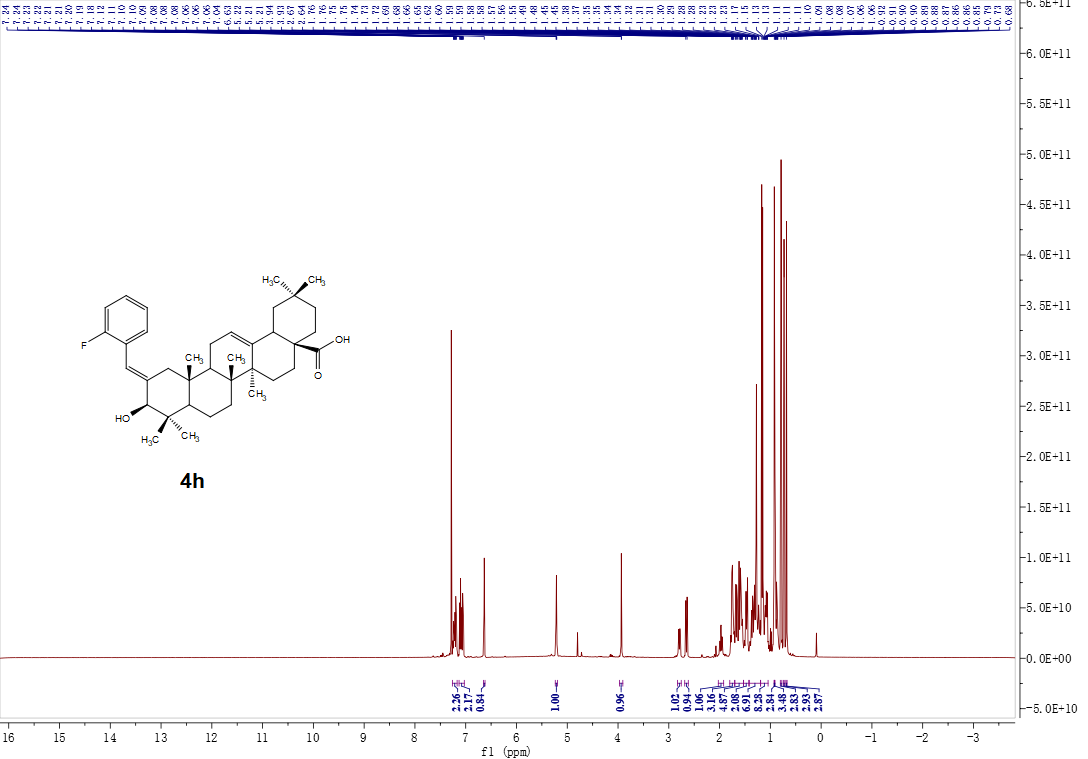


**Fig.S15** 4h **(**^1^H NMR)

**Fig.S16** 4**h (**^13^C NMR)


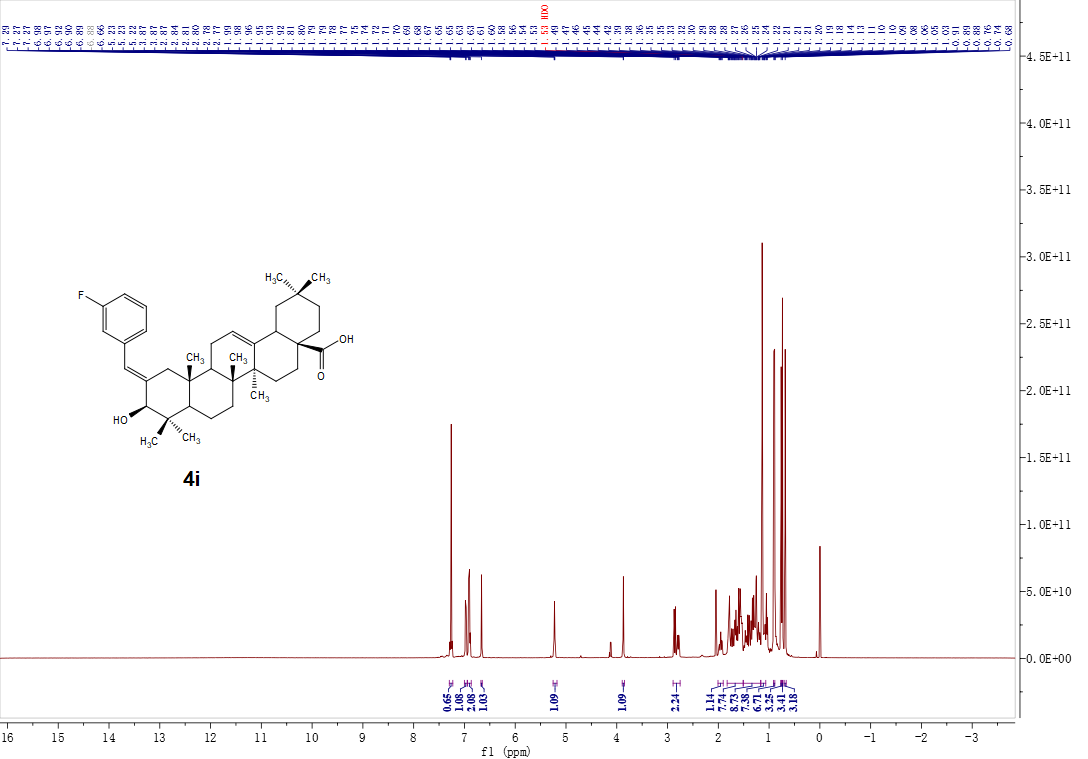


**Fig.S17** 4**i (**^1^H NMR)

**Fig.S18** 4**i (**^13^C NMR)


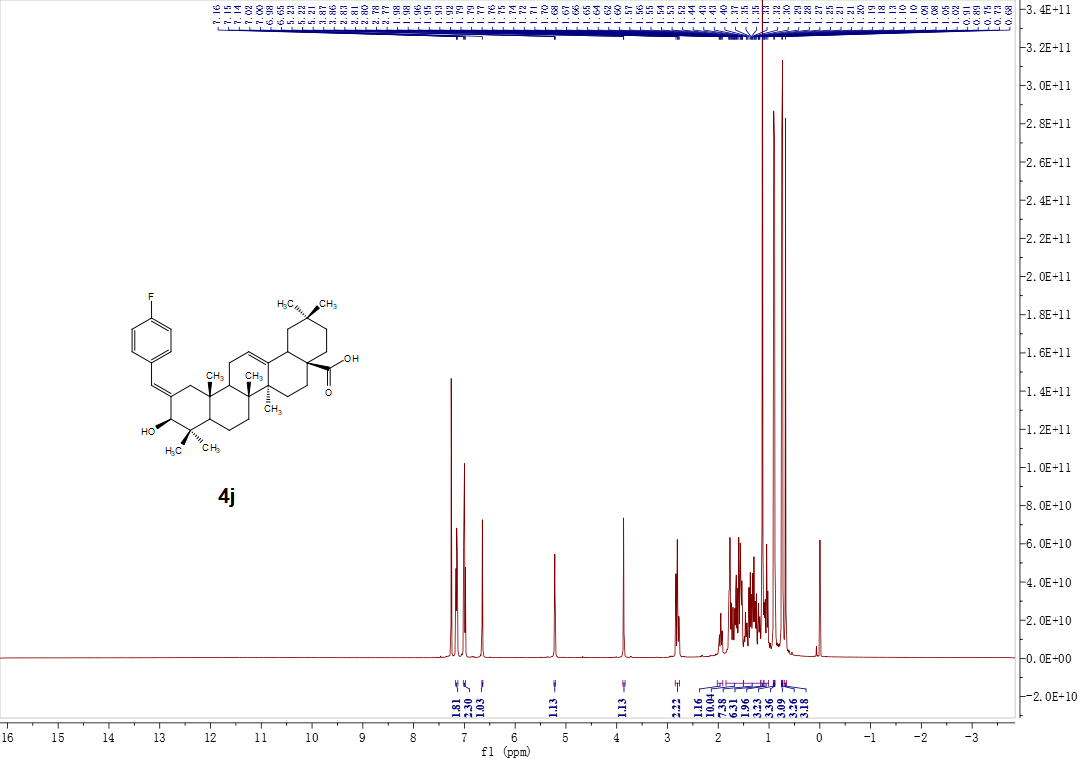


**Fig.S19** 4j **(**^1^H NMR)


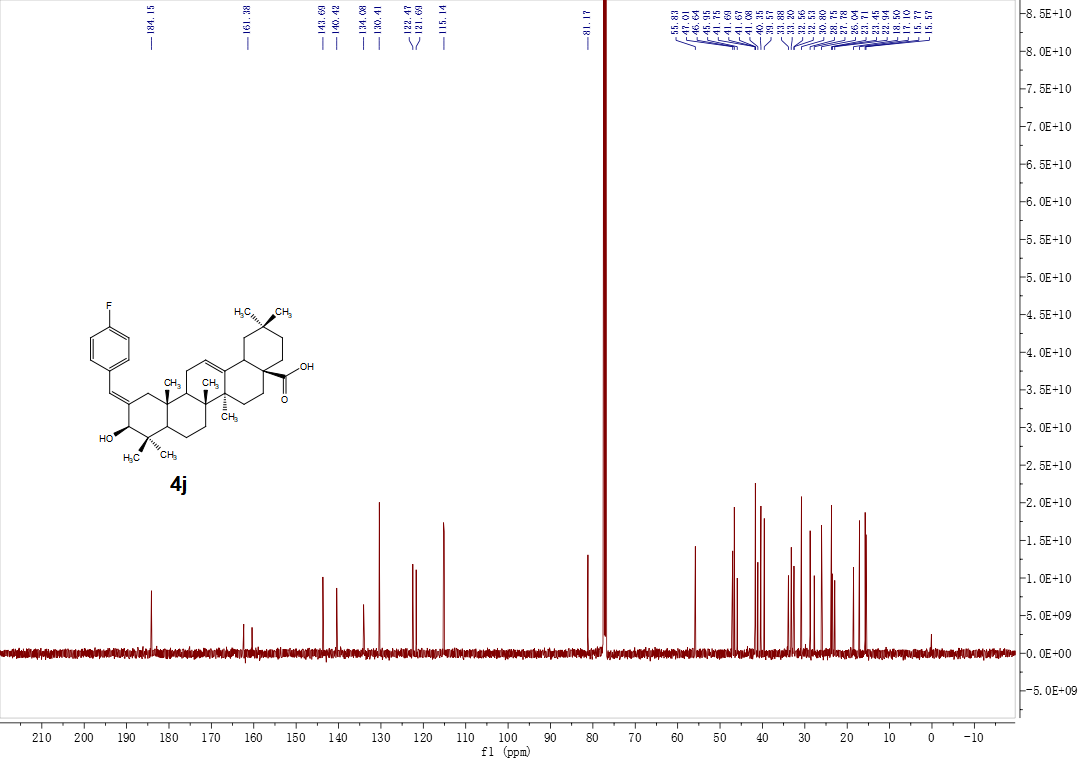


**Fig.S20** 4**j (**^13^C NMR)


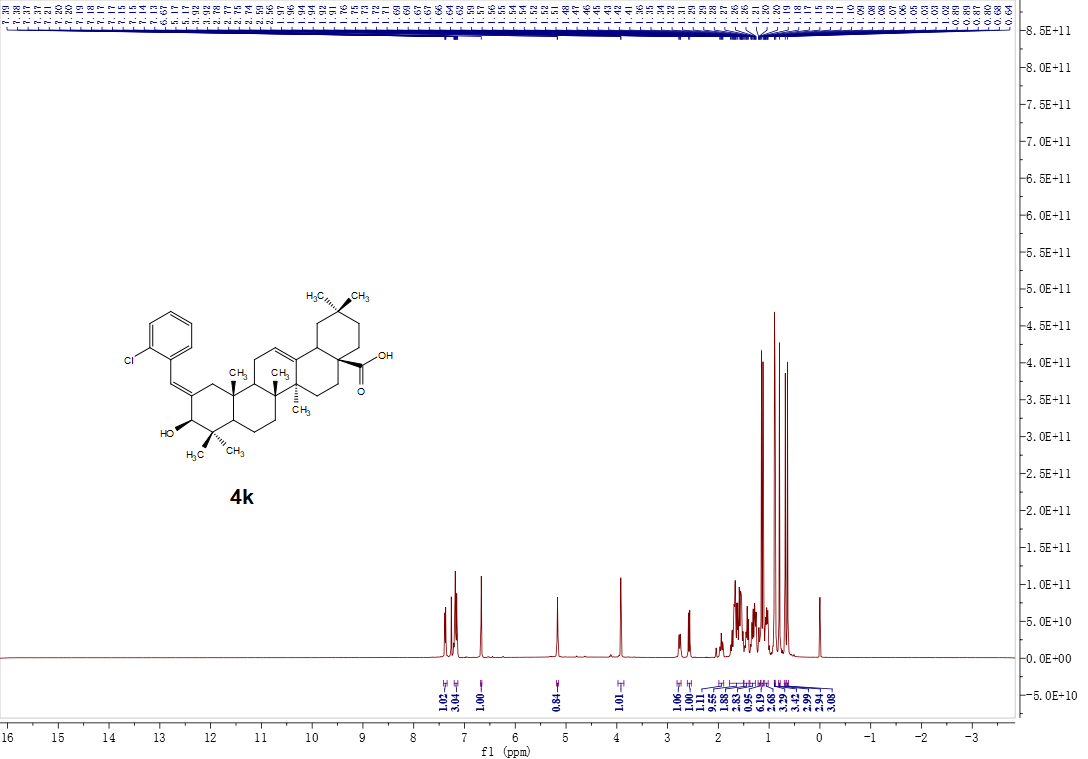


**Fig.S21** 4k **(**^1^H NMR)

**Fig.S22** 4**k (**^13^C NMR)

**Fig.S23** 4l **(**^1^H NMR)

**Fig.S24** 4l **(**^13^C NMR)


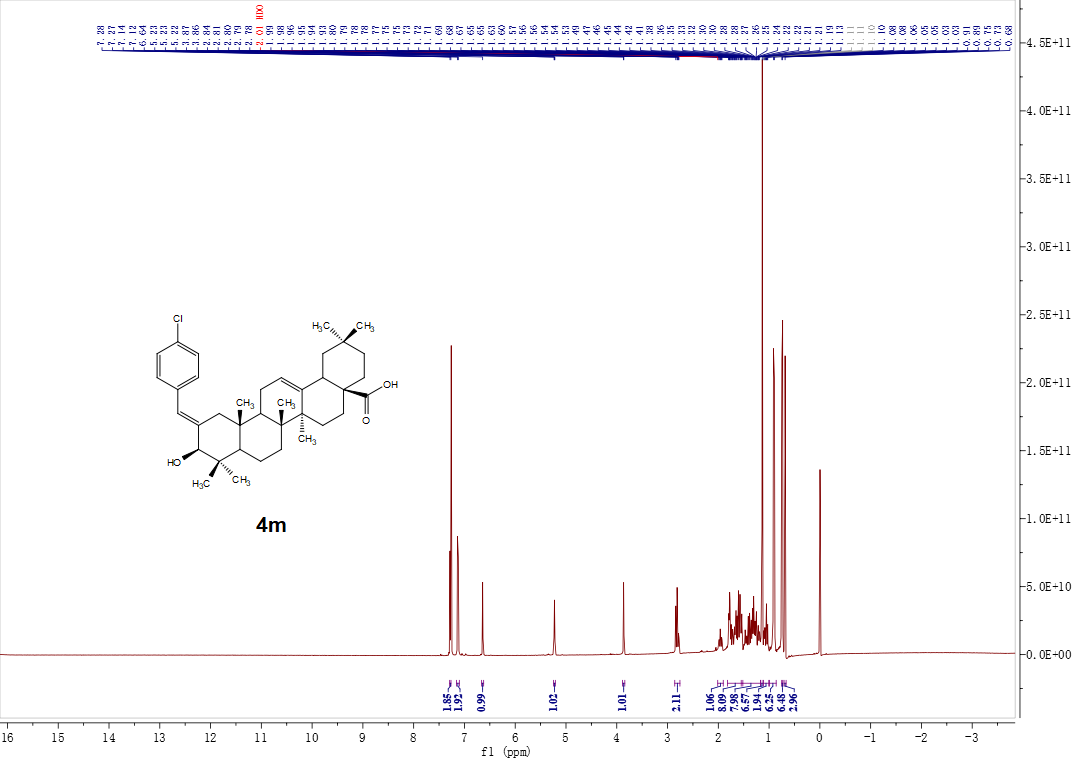


**Fig.S25** 4m **(**^1^H NMR)

**Fig.S26** 4m **(**^13^C NMR)


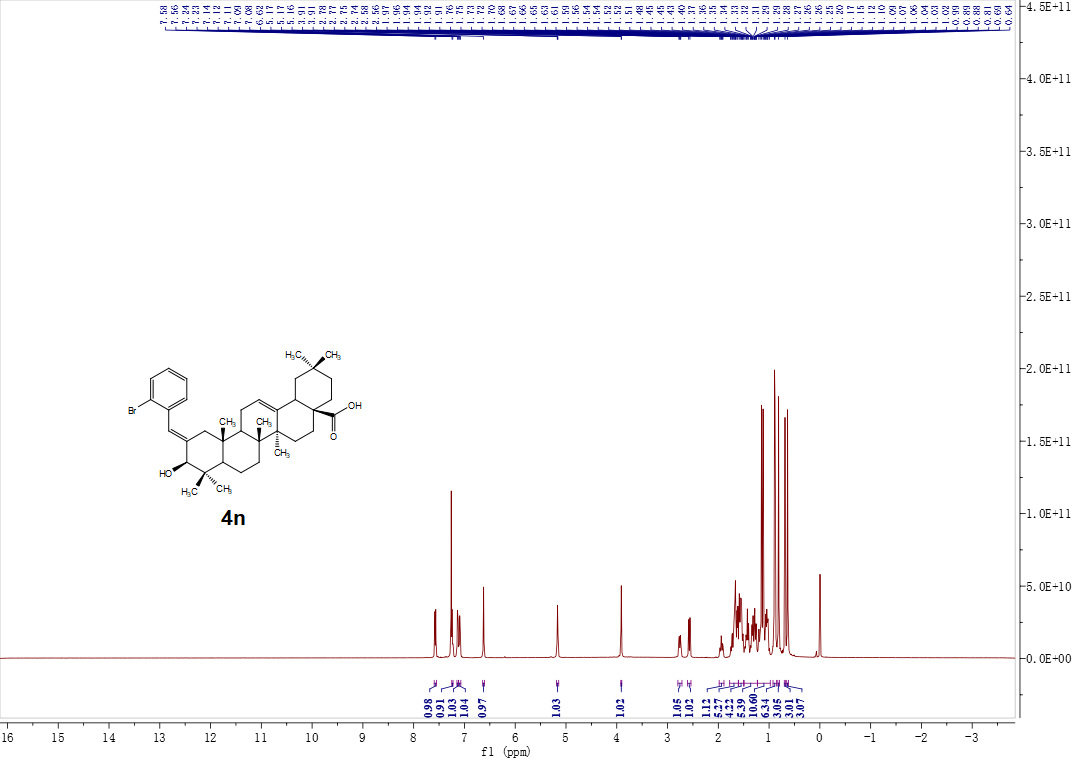


**Fig.S27** 4n **(**^1^H NMR)

**Fig.S28** 4n **(**^13^C NMR)

**Fig.S29** 4o **(**^1^H NMR)

**Fig.S30** 4o **(**^13^C NMR)


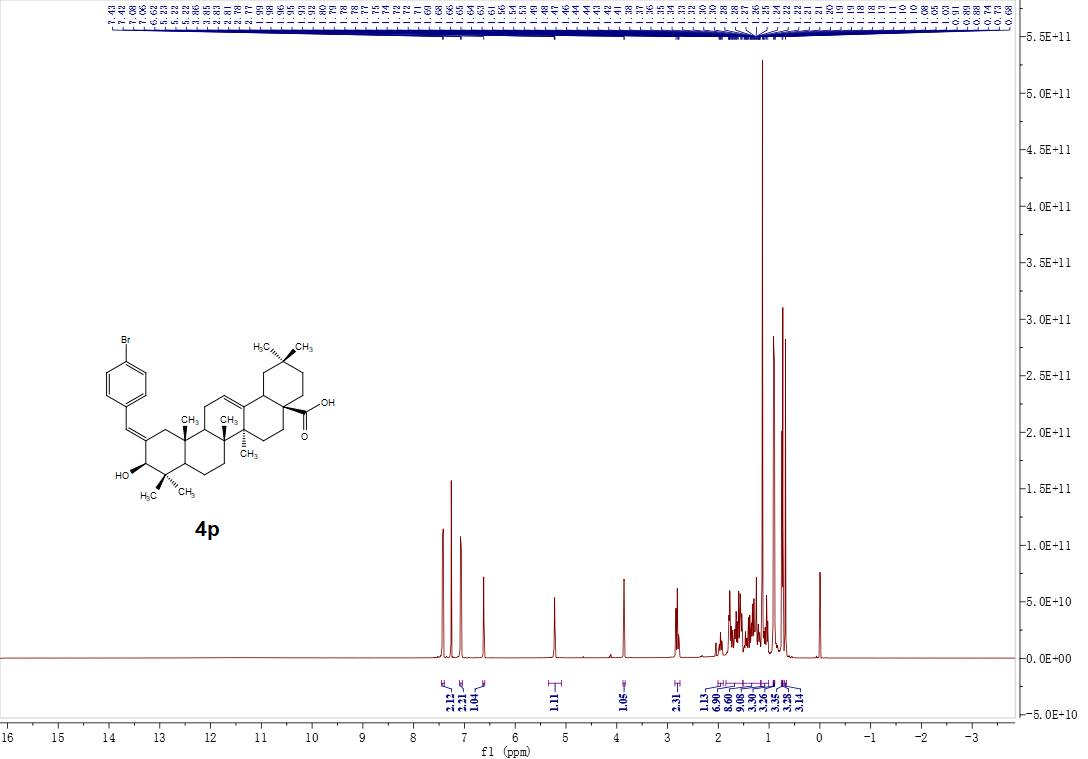


**Fig.S31** 4p **(**^1^H NMR)

**Fig.S32** 4**p(**^13^C NMR)


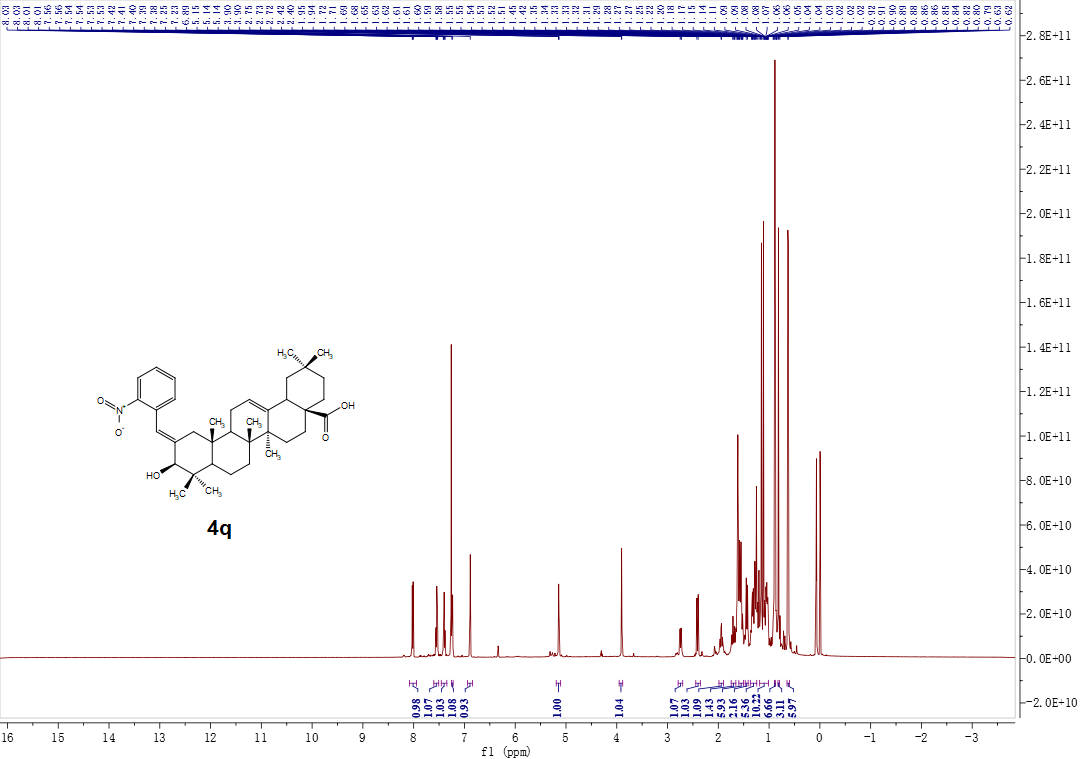


**Fig.S33** 4**q(**^1^H NMR)

**Fig.S34** 4**q (**^13^C NMR)

**Fig.S35** 4**r (**^1^H NMR)

**Fig.S36** 4**r (**^13^C NMR)

**Fig.S37** 4**s (**^1^H NMR)

**Fig.S38** 4**s (**^13^C NMR)

**Fig.S39** 4**a (**HRMS)

**Fig.S40** 4**b (**HRMS)

**Fig.S41** 4**c (**HRMS)

**Fig.S42** 4**d (**HRMS)

**Fig.S43** 4**e (**HRMS)

Fig.44 4**f (**HRMS)

**Fig.S45** 4**g (**HRMS)

**Fig.S46** 4**h (**HRMS)

**Fig.S47** 4**i (**HRMS)

**Fig.S48** 4**j (**HRMS)

**Fig.S49** 4**k (**HRMS)

**Fig.S50** 4**l (**HRMS)

**Fig.S51** 4**m (**HRMS)

**Fig.S52** 4**n (**HRMS)

**Fig.S53** 4**o (**HRMS)

**Fig.S54** 4**p (**HRMS)

**Fig.S55** 4**q (**HRMS)

**Fig.S56** 4**r (**HRMS)

**Fig.S57** 4**s (**HRMS)


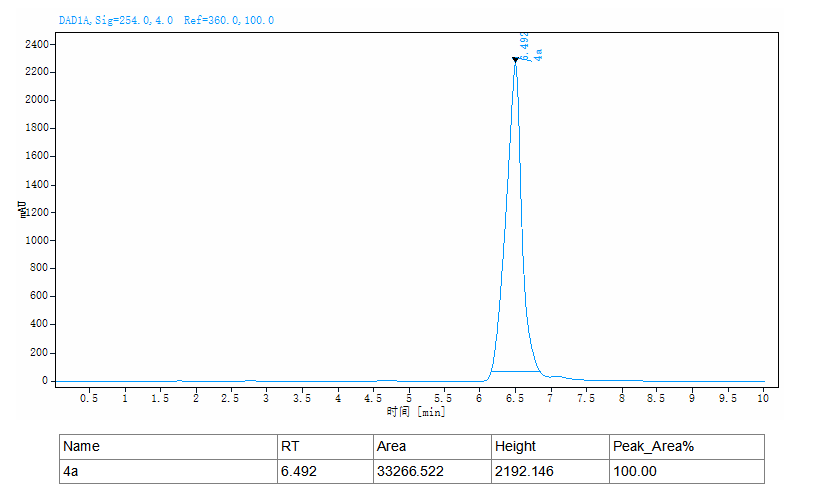


**Fig.S58 4a (**HPLC)


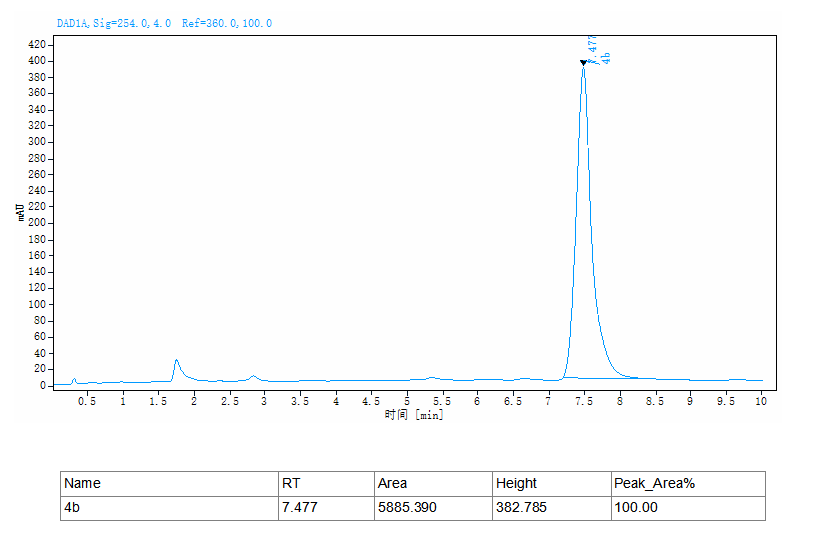


**Fig.S59 4b (**HPLC)


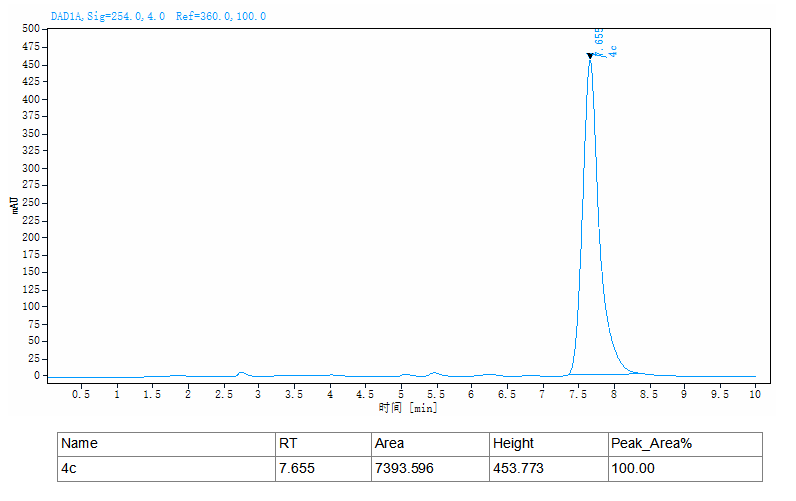


**Fig.S60** **4c** (HPLC)


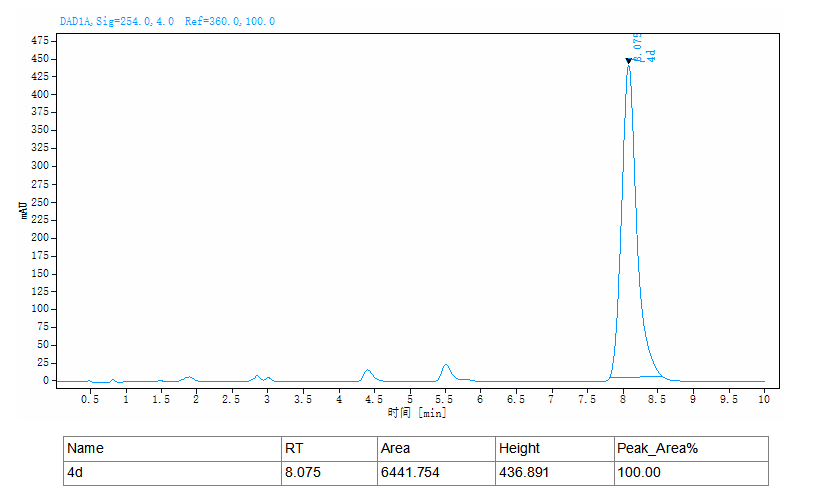


**Fig.S61** **4d (**HPLC)


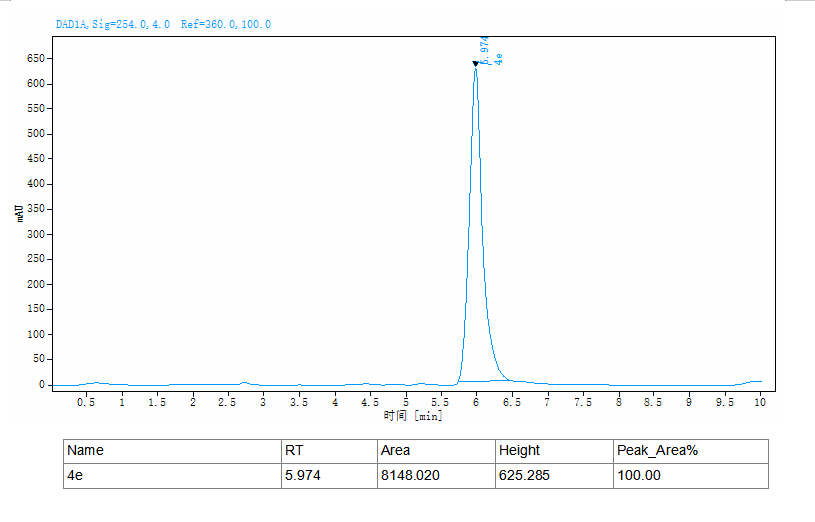


**Fig.S62** **4e (**HPLC)


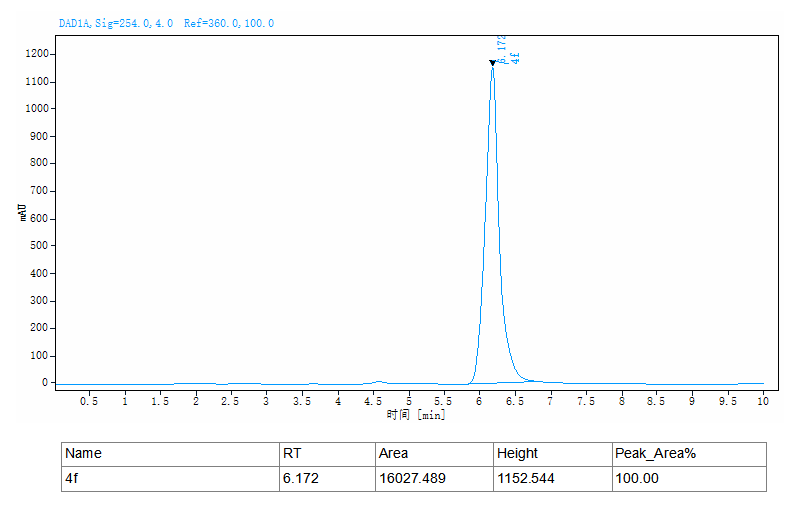


**Fig.S63** **4f (**HPLC)


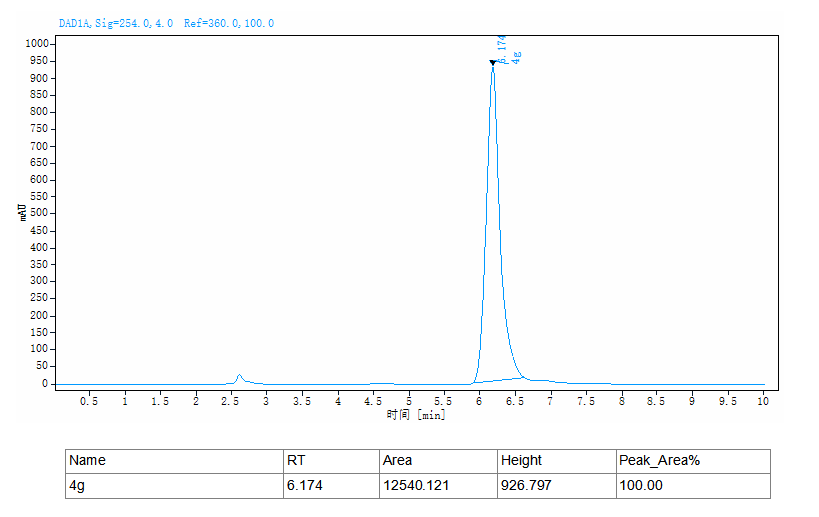


**Fig.S64 4g** **(**HPLC)


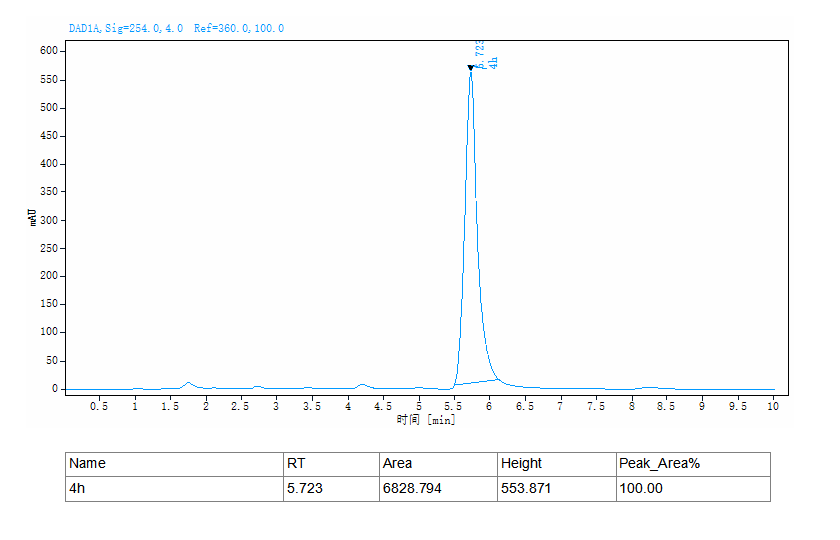


**Fig.S65** **4h** **(**HPLC)


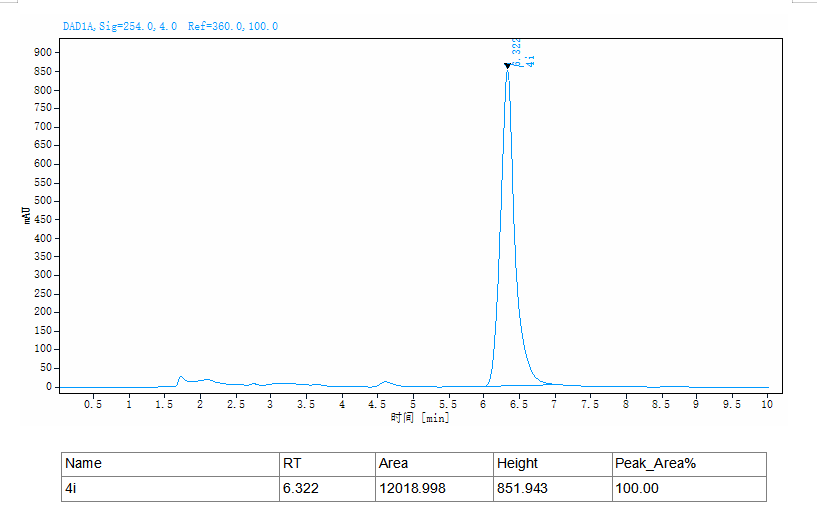


**Fig.S66** **4i** **(**HPLC)


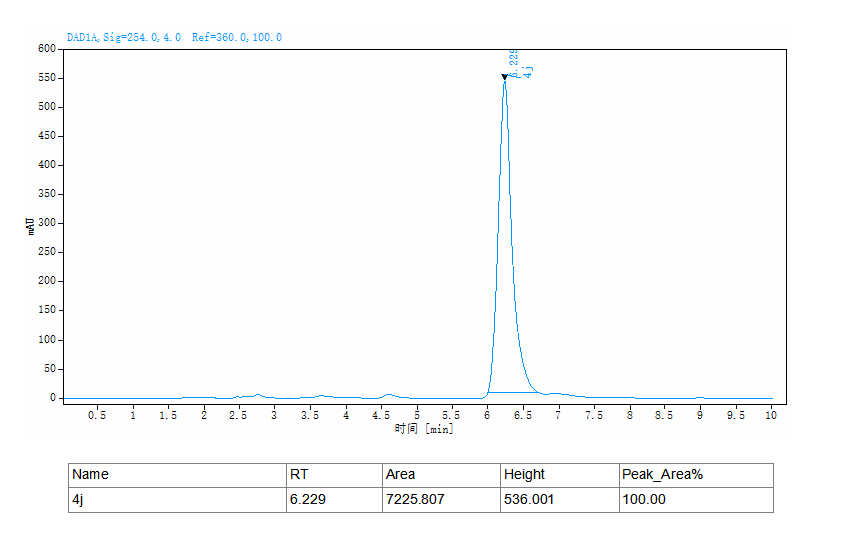


**Fig.S67** **4j** **(**HPLC)


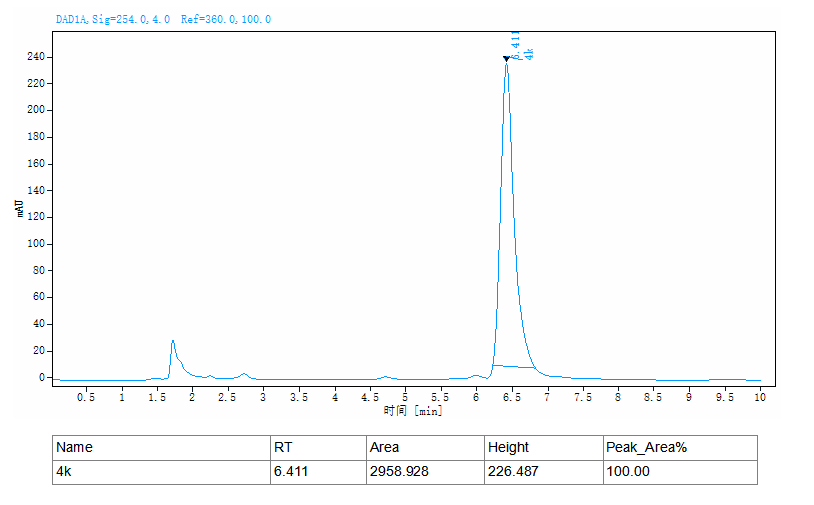


**Fig.S68** **4k** **(**HPLC)


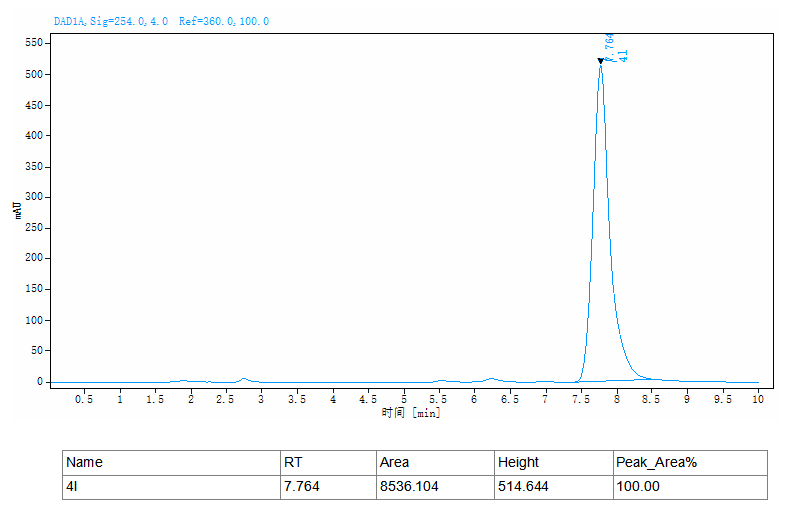


**Fig.S69** **4l (**HPLC)


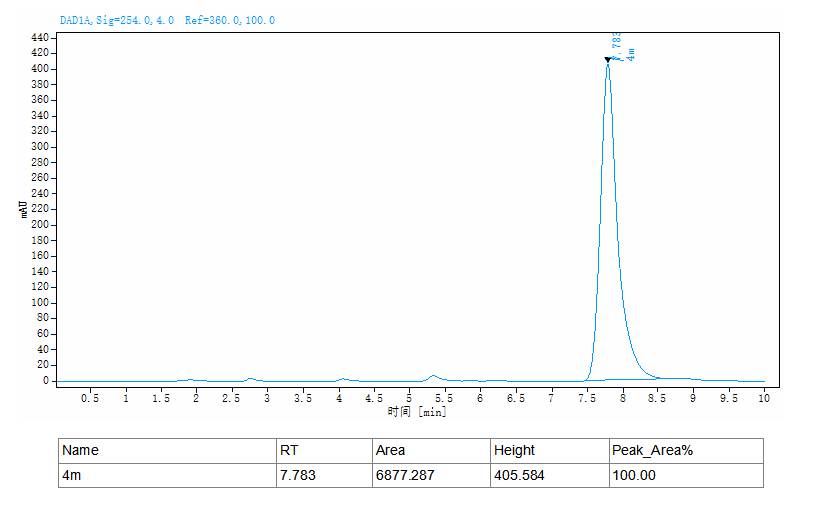


**Fig.S70** **4m** **(**HPLC)


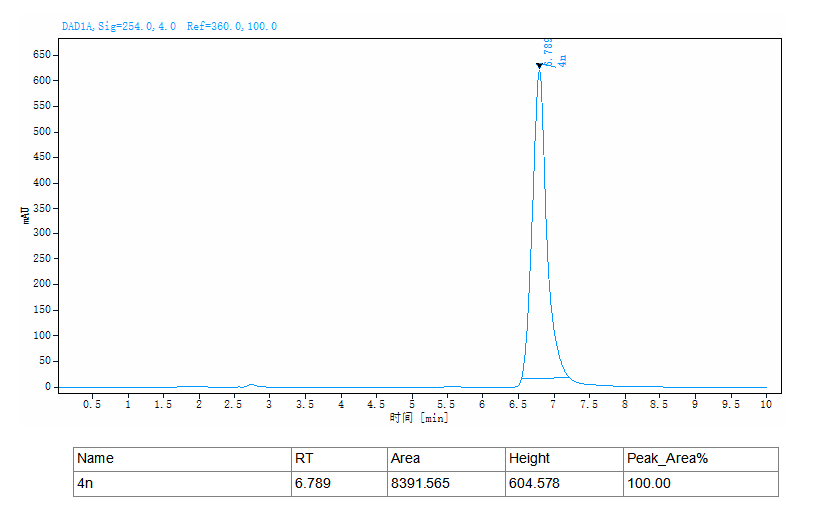


**Fig.S71 4n** **(**HPLC)


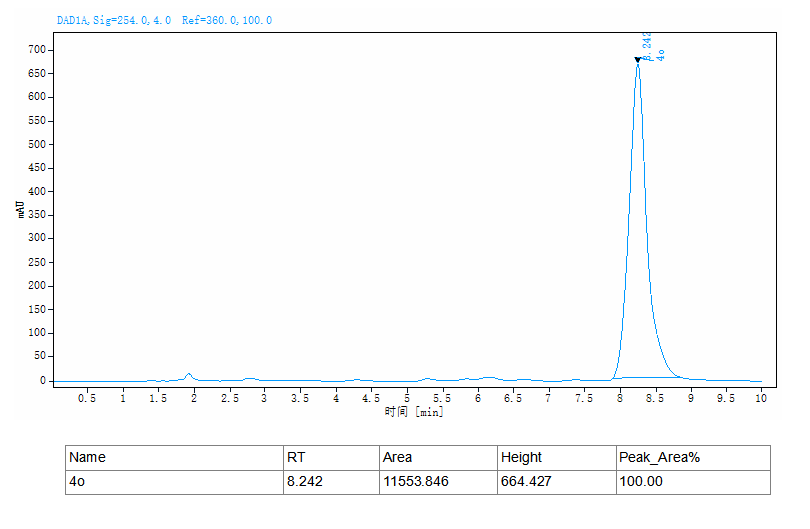


**Fig.S72 4o (**HPLC)


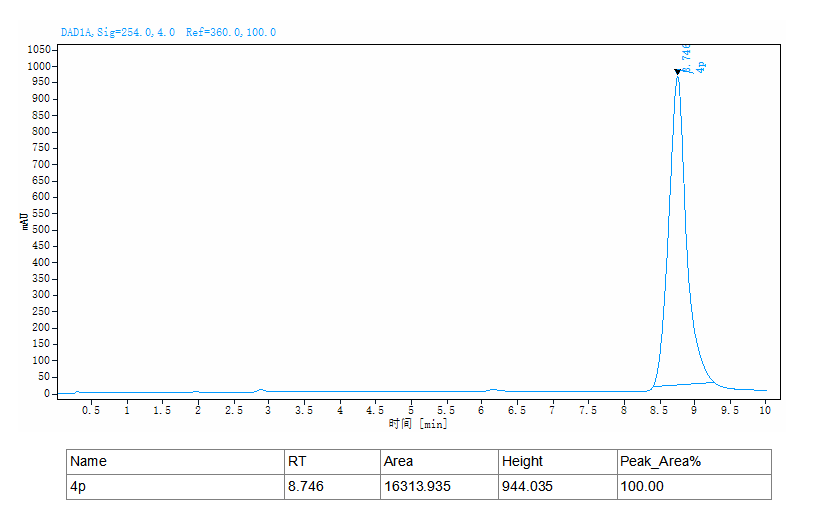


**Fig.S73** **4p** **(**HPLC)


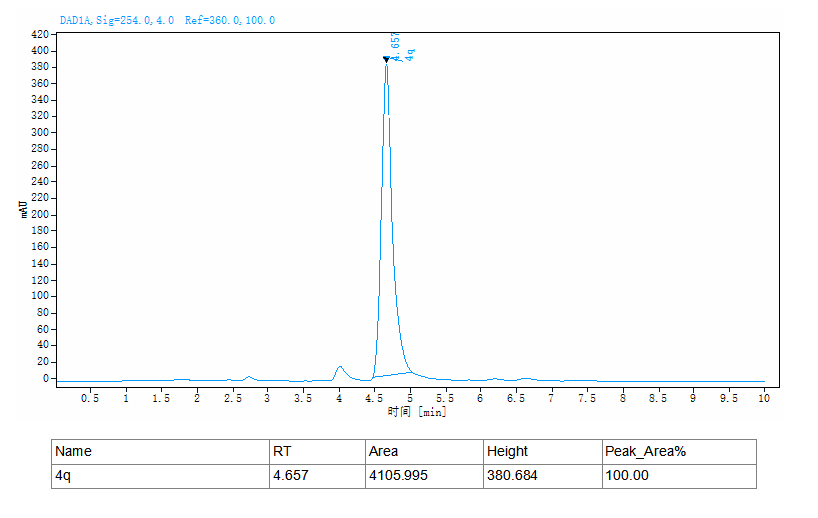


**Fig.S74** **4q** **(**HPLC)


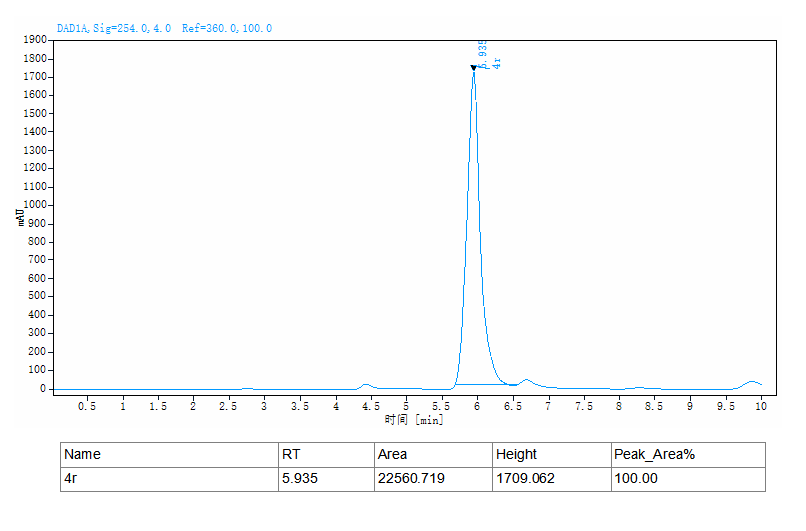


**Fig.S75** **4r (**HPLC)


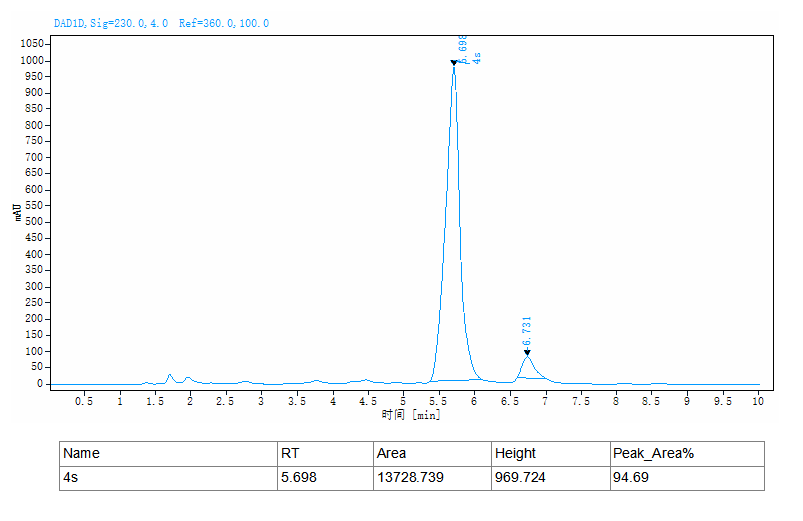


**Fig.S76 4s** **(**HPLC)

**Fig.S77** The inhibition curves for compounds **4i** against α-glucosidase

**Fig.S78** The inhibition curves for compounds **4o** against *α*-amylase
